# Atypical electrophysiological and behavioral responses to diazepam in a leading mouse model of Down syndrome

**DOI:** 10.1038/s41598-021-89011-y

**Published:** 2021-05-04

**Authors:** Daniella B. Victorino, Daniel J. L. L. Pinheiro, Jonah J. Scott-McKean, Sarah Barker, Melissa R. Stasko, Jean Faber, Carla A. Scorza, Alberto C. S. Costa

**Affiliations:** 1 Discipline of Neuroscience, Department of Neurology and Neurosurgery, Federal University of São Paulo, Paulista Medical School, São Paulo, SP Brazil; 2 Division of Pediatric Neurology, Department of Pediatrics, Case Western Reserve University, 11100 Euclid Avenue, Mail Stop RBC 6090, Cleveland, OH 44106-6090 USA; 3 Department of Psychiatry, Case Western Reserve University, Cleveland, OH USA

**Keywords:** Diseases of the nervous system, Developmental disorders, Autism spectrum disorders

## Abstract

Mounting evidence implicates dysfunctional GABA_A_R-mediated neurotransmission as one of the underlying causes of learning and memory deficits observed in the Ts65Dn mouse model of Down syndrome (DS). The specific origin and nature of such dysfunction is still under investigation, which is an issue with practical consequences to preclinical and clinical research, as well as to the care of individuals with DS and anxiety disorder or those experiencing seizures in emergency room settings. Here, we investigated the effects of GABA_A_R positive allosteric modulation (PAM) by diazepam on brain activity, synaptic plasticity, and behavior in Ts65Dn mice. We found Ts65Dn mice to be less sensitive to diazepam, as assessed by electroencephalography, long-term potentiation, and elevated plus-maze. Still, diazepam pre-treatment displayed typical effectiveness in reducing susceptibility and severity to picrotoxin-induced seizures in Ts65Dn mice. These findings fill an important gap in the understanding of GABAergic function in a key model of DS.

## Introduction

Down syndrome (DS), the phenotypic expression of trisomy 21, accounts for approximately 1 in 700 live births in the United States and has a prevalence of about 1 in 1000 in the general population^1^. Persons with DS are especially vulnerable to neurodevelopmental and neurodegenerative disorders. The intellectual disability displayed by individuals with DS is mostly generalized, with disproportionate involvement of abilities heavily dependent on the hippocampus and prefrontal cortex^2,3^. Trisomy 21 also affects the central nervous system (CNS) in several additional ways. For example, persons with DS are at a higher risk of experiencing seizure and anxiety disorders than those in the general population^4–10^.

The idea that the inhibition of gamma-aminobutyric acid (GABA) A receptors (GABA_A_R) could restore long-term potentiation (LTP) in hippocampal slices from Ts65Dn mice has been explored by Kleschevnikov and collaborators^11^ and members of our research team^12,13^. However, in these studies, the doses necessary to produce rescuing of the observed depressed levels of LTP in Ts65Dn mice were convulsive doses that generated robust network oscillations in hippocampal slice preparations^12^. Subsequent work^14^, however, showed that Ts65Dn mice treated with a subconvulsive dose of picrotoxin, once a day for 2 weeks, displayed a durable enhancement in object recognition performance and in dentate-gyrus LTP. Although the mechanisms underlying this observation remain poorly understood, the idea that modulating the synaptic balance between inhibition and excitation could become a practical therapeutic approach in individuals with DS became a popular concept for several years. This idea even inspired the design and implementation of a few small-scale clinical trials with either the GABA_A_ receptor antagonist pentylenetetrazole (PTZ or BTD-001^15^) or the selective GABAA α5 receptor negative allosteric modulator (RG1662 or Basmisanil^16^^,^^17^). Fortunately, these trials concluded without any severe adverse events among the participants from this vulnerable population, and the underlying idea of inhibiting GABA_A_R-mediated neurotransmission as a potential therapeutic strategy in DS has mostly fallen out of favor in recent years.

Recently, a completely different perspective on GABA_A_R-mediated neurotransmission dysfunction in DS was brought into view by the work by Deidda et al.^18^, which generated extensive evidence supporting the notion that intracellular accumulation of chloride ions (Cl^−^) occurs in the hippocampus of Ts65Dn mice. According to these authors, this phenomenon would result from an increased expression of the cation-Cl^−^ co-transporter NKCC1, which would lead to the reversal of GABA_A_R responses from inhibitory (hyperpolarization) to excitatory (depolarization)^18^. One of the predicted consequences of such alteration in Cl^−^ homeostasis in Ts65Dn mice would be that molecules that act by enhancing GABA-mediated responses might not be as effective in reducing neuronal activity during pathologic synchronization events, or might even flip inhibition into paradoxical excitation on this mouse model (and presumably in individuals with DS). Of particular concern would be benzodiazepines, which are very popular anxiolytic/anti-seizure agents that positively modulate GABAergic inhibitory neurotransmission by increasing the frequency of channel opening of GABA_A_R in response to GABA^19^.

In terms of clinical practice, there would be several areas of DS-specific concerns regarding the use of benzodiazepines in the event of altered brain Cl^−^ homeostasis. For example, considering that benzodiazepines are the first-line treatment of acute seizure disorders, should we reconsider the use of such agents for treating emergent seizures in individuals with DS^20^? Additionally, because young and older adults with DS display increased vulnerability to generalized anxiety disorder, and benzodiazepines are also among the most effective interventions in patients with DS with clinical signs of catatonia and unexplained regression^5,10,21^, do we need to re-evaluate how we approach the treatment of these psychiatric disorders in persons with DS?

In the present study, we evaluated the effects of the highly prescribed benzodiazepine diazepam on the electrophysiological and behavioral phenotypes of the most-widely used mouse model of DS, the Ts65Dn mouse. To this end, we used electroencephalography (EEG) to investigate how diazepam modulates local field potential (LFP) activity in the cortex and hippocampus of awake, freely-behaving Ts65Dn mice. We also quantified the effects of diazepam and the neurotransmitter GABA itself on LTP in the CA1 region of Ts65Dn mouse hippocampus. Finally, we evaluated mouse behavior in the elevated plus-maze (EPM) task to probe for differences between Ts65Dn and control mice in their responsiveness to the anxiolytic-like properties of diazepam. Although our findings did not support the notion of diazepam-induced enhanced excitability, this study unveiled significant differences in the effects of diazepam on GABA_A_R-mediated responses between control and Ts65Dn mice. Such findings should inspire future preclinical and clinical studies in DS and many other neurodevelopmental, psychiatric, and neurological disorders in which an alteration in Cl^−^ homeostasis is suspected^22^.

## Results

### GABA_A_R modulation by diazepam does not cause clinical or electrographic seizures in Ts65Dn mice

**Figure 1:**
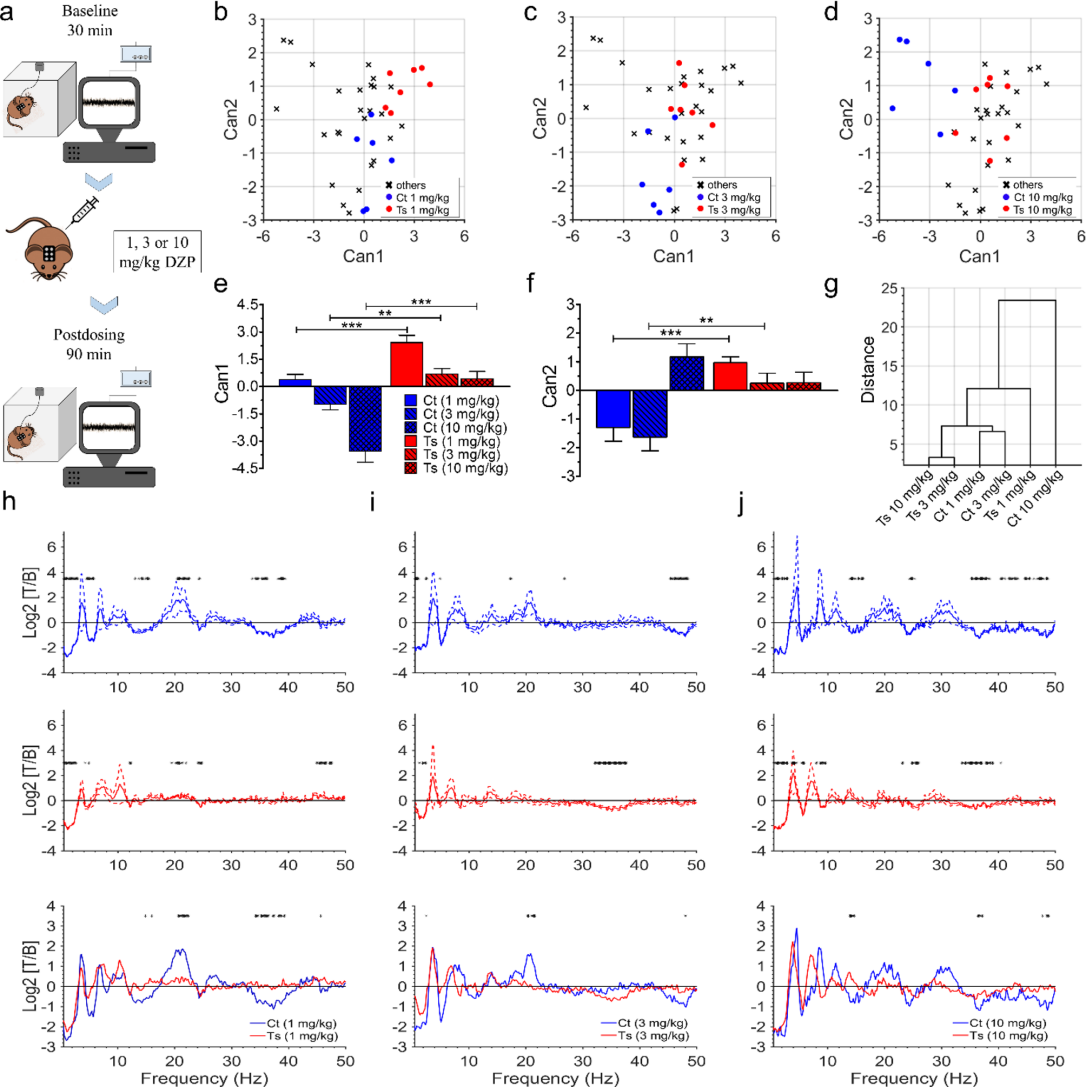
Diazepam induces significantly different changes on the cortical activity spectral profile of control and Ts65Dn mice. (**a**) Freely moving mice implanted with EEG electrodes were connected through a cable to the recording unit. The experiment consisted of a 30-min baseline recording, followed by a 90-min post-dosing recording, in which mice were acutely treated with 1, 3, or 10 mg/kg diazepam (i.p.). (**b**–**d**) Grouped scatter plots of the first two canonical variables for all mice analyzed are shown in (**b**–**d**) for 1-, 3-, and 10-mg/kg doses of diazepam, respectively. Colored points represent mice and are labelled by cluster group. (**e**) *Can1* discriminated the control mice group from the Ts65Dn mice group at 1-, 3-, and 10-mg/kg doses. (**f**) *Can2* differentiated control and Ts65Dn mice groups at 1- and 3-mg/kg doses. (**g**) The centroid of control mice group treated with 10 mg/kg diazepam was at the highest level of the dendrogram’s hierarchical tree, whereas the centroids of Ts65Dn mice groups treated with 3- and 10-mg/kg doses had the shortest distance among all centroids. (**h**–**j**) PSD obtained from baseline and post-dosing recordings were compared for each dose in each genotype (black asterisks in upper and middle panels represent frequency bins in which significant differences in EEG spectral power were observed between baseline and treatment conditions; relative changes are expressed as the log_2_ of the treatment/baseline ratio). Normalized PSD of control and Ts65Dn mice for each experimental condition was also compared (lower panels). Number of mice: Ct (N = 6) and Ts (N = 7) for all experimental conditions. In (**e**, **f**), data represent mean ± SEM and statistical significance is expressed as ** and *** for *P* < 0.01 and *P* < 0.001, respectively. In (**h**–**j**), solid lines represent means and dashed lines represent SEM and black asterisks indicate statistical significance (*P* < 0.05).

To determine the behavioral effects of diazepam on Ts65Dn (N = 15) and control (N = 14) mice (13–20 weeks old), video recordings were performed in freely moving mice acutely treated with 1, 3, or 10 mg/kg diazepam (Fig. 1a). Subsets of Ts65Dn (N = 7) and control (N = 6) animals were chronically implanted with EEG electrodes to record brain electrical activity during these video recording sessions (video-EEG).

Before diazepam injections, mice were freely moving around their cages or standing along the cage sides. After injection, all diazepam doses produced relaxed wakefulness or drowsiness in both control and Ts65Dn mice. Diazepam-treated mice no longer showed a preference for the periphery of their cages and they frequently laid in a stretched-out posture (instead of curled-up) or sat quietly in any cage location. Mice eyelids were partially or fully open following diazepam administration. Visual inspection of the video-EEG recordings revealed that Ts65Dn and control mice showed no evidence of electrographic seizures or epilepsy-related phenotypes, neither spontaneously nor pharmacologically induced by any of the administered doses of diazepam.

To estimate the effects of diazepam on neuronal activity, we first calculated the power-spectrum distribution (PSD) over the 0.5–100-Hz frequency range in the video-EEG subset of animals. A normalized PSD was obtained for each experimental condition by the ratio of the PSDs for the EEG 30-min post-dosing segments and those for the EEG 30-min baseline-recording segments. Quantitatively similar results were observed for the EEG spectral analysis of cortical and hippocampal recordings. Thus, we will only report EEG data obtained from the cortical derivation in the main text (the EEG analysis dataset from the hippocampal derivation can be found in Supplementary Figs. S1 and S2 and Supplementary Tables S1–S5). Given that we did not observe major differences between control and Ts65Dn mice in EEG power across frequencies from 50 to 100 Hz, we will mostly focus on comparisons between EEG power across the frequency range of 0.5–50 Hz (results of spectral parameters within the high-gamma band can be found in Supplementary Tables S3 and S4).

We employed one-way MANOVA followed up with canonical discriminant analysis (*Can*) to test whether changes in EEG spectral power across multiple 2-Hz bands (0.5–50 Hz) induced by different doses of diazepam could be used to differentiate control from Ts65Dn mice (Fig. 1b–d). Visual inspection of the grouped scatter plots of the first two canonical variables showed that *Can1* discriminated the control mice group from the Ts65Dn mice group at 1-, 3-, and 10-mg/kg doses (Fig. 1b–d respectively). One-way ANOVA (F_(5,33)_ = 25.590, *P* < 0.0001), followed by Fisher’s LSD post hoc test (*P* = 0.0009, *P* = 0.0055, and *P* < 0.0001 at 1-, 3-, and 10-mg/kg doses, respectively), confirmed these findings (Fig. 1e; Supplementary Tables S1 and S2). Similarly, *Can2* differentiated control and Ts65Dn mice groups (F_(5,33)_ = 8.346, *P* < 0.0001) at 1- and 3-mg/kg doses (*P* = 0.0003 and *P* = 0.0019, respectively; Fig. 1f; Supplementary Tables S1 and S2). It is important to emphasize that the relative size of *Can1* (58.14%) is much larger than *Can2* (18.96%) or any of the calculated higher order canonical variables (Fig. S2).

We also evaluated similarities among groups by using hierarchical clustering analysis. A dendrogram representation of the Euclidian distances between group centroids (i.e., the group's mean), showed for example that the centroid of control mice group treated with 10 mg/kg diazepam was at the highest level of the hierarchical tree (Fig. 1g). The full set of observed inter-cluster differences in the grouped scatter plots suggest a genotype-dependence for the effects of diazepam on EEG spectral power. This led us to compare the normalized PSDs obtained from baseline and post-dosing recordings for each dose in each genotype (Fig. 1h–j, upper and middle portions) to determine the differences in diazepam effects between control and Ts65Dn mice for each experimental condition (Fig. 1h–j, lower portions).

We observed that the effects of diazepam on slower brain rhythms were mildly different between control and Ts65Dn mice (Fig. 1h–j). Spectral power decreases in the 0.50–2.90 Hz and 4.50–5.70 Hz ranges were induced by 1 mg/kg diazepam in control mice (Fig. 1h, upper portion), whereas in Ts65Dn mice this decrease in power only occurred in the 0.50–2.70 Hz range (Fig. 1h, middle portion). Additionally, spectral power reductions were restricted to narrower frequency bands at the 3-mg/kg dose in both genotypes (0.50–1.10 Hz and 1.95–2.45 Hz in control and Ts65Dn mice, respectively; Fig. 1i, upper and middle portions). Finally, power reductions induced by 10-mg/kg doses occurred at the frequency ranges of 0.50–2.90 Hz (Fig. 1j, upper portion) in control mice and at 1.20–2.90 Hz and 8.80–9.50 Hz (Fig. 1j, middle portion) for Ts65Dn mice.

The presence of increased beta activity following PAM of GABA_A_Rs has been widely recognized as an EEG biomarker in both animal models^23,24^ and humans^25,26^. As shown in Fig. 1h (upper portion), we detected a consistent power increase affecting the frequency range of 20.50–22.60 Hz in control mice treated with 1 mg/kg diazepam, which did not reach significance for the 3 and 10-mg/kg doses (Fig. 1h,j, upper portions). In contrast, we did not find evidence of power increase within beta-frequency range in Ts65Dn mice treated with any dose of diazepam (Fig. 1h–j, middle portions). In addition, we detected significant differences in the spectral profile of beta activity between control and Ts65Dn mice at both 1 and 3 mg/kg diazepam (Fig. 1h,i, lower portion). Finally, significant differences in the spectral profile of gamma activity (34.15–37.35 Hz) were detected between control and Ts65Dn mice at 1-mg/kg dose (Fig. 1h, lower portion).

Given that 1-mg/kg was more effective than 3- and 10-mg/kg doses in eliciting a measurable physiological effect in cortical network oscillations, we will focus on the EEG effects of 1 mg/kg diazepam in the following analyses (results of the EEG parameters for 3 and 10 mg/kg diazepam can be found in Supplementary Tables S3–S5). Furthermore, 1-mg/kg dose in mice can be considered equivalent to a ~ 5 mg dose in humans (using 60 kg as body weight reference^27^), which is in the middle range of the common diazepam doses used in clinical practice (2–10 mg^28^).

### For each frequency band, we observed genotype-dependent changes in neuronal activity in response to 1 mg/kg diazepam

We analyzed a variety of spectral features extracted from the PSD to further explore the effects of diazepam at 1-mg/kg dose on GABAergic transmission mediated by GABA_A_R. In this set of analysis, the PSD was decomposed into the functionally distinct wavebands delta (0.5–4 Hz), theta (4–8 Hz), alpha (8–12 Hz), beta 1 (12–15 Hz), beta 2 (15–30 Hz), and gamma (30–50 Hz). We also calculated the mean frequency power for each waveband. (Analysis of the pattern of the spectral power distribution across frequency bands can be found in Supplementary Tables S3–S5).

**Figure 2:**
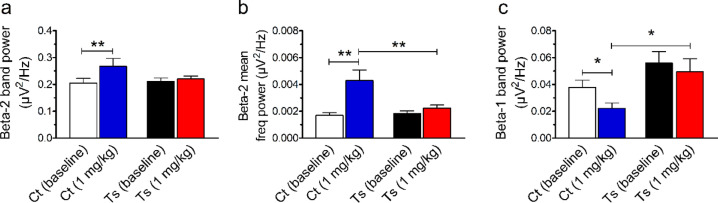
Ts65Dn mice show altered beta-band neuronal activity responses when compared to control euploid mice. (**a**) Diazepam at 1-mg/kg dose significantly increased beta-2 band power in control but not in Ts65Dn mice. (**b**) Diazepam-treated control mice showed an increase in the power of the beta-2 mean frequency compared to its baseline condition and diazepam-treated Ts65Dn mice. (**c**) Diazepam-treated control mice also showed a lower beta-1 band power than its baseline condition and diazepam-treated Ts65Dn mice. Number of mice: Ct baseline (N = 6), Ct 1 mg/kg diazepam (N = 6), Ts baseline (N = 7), and Ts 1 mg/kg diazepam (N = 7). Data represent mean ± SEM. Statistical significance is expressed as * and ** for *P* < 0.05 and *P* < 0.01, respectively.

We observed that the effects of diazepam on EEG spectral power were dependent on frequency band. Diazepam at 1-mg/kg dose significantly (F_(1, 11)_ = 9.472, *P* = 0.0105) increased beta-2 band power in control but not in Ts65Dn mice. A significant interaction was also found between genotype and treatment (F_(1, 11)_ = 5.307, *P* = 0.0418). Post hoc tests confirmed that diazepam-treated control mice showed a significantly higher beta-2 band power compared with its baseline condition (*P* = 0.0037; Fig. 2a). In addition, we found significant treatment- and genotype-dependent effects (F_(1, 11)_ = 12.268, *P* = 0.0049 and F_(1, 11)_ = 6.323, *P* = 0.0288; respectively) on the beta-2 mean frequency power. A significant interaction between genotype and treatment was also detected (F_(1, 11)_ = 6.537, *P* = 0.0267), with diazepam-treated control mice showing a significant increase in the power of the beta-2 mean frequency compared to its baseline condition (*P* = 0.0017) and to diazepam-treated Ts65Dn mice (*P* = 0.0017; Fig. 2b). Such findings support our previous observation that diazepam increases EEG activity within the beta-2 frequency range in a genotype-dependent manner.

We also found that diazepam at 1-mg/kg dose decreased beta-1 band power in control but not in Ts65Dn mice. Both genotype and treatment had significant effects (F_(1, 11)_ = 5.350, *P* = 0.0411 and F_(1, 11)_ = 5.496, *P* = 0.0389; respectively), with detected decreases in the beta-1 band power compared to its baseline condition (*P* = 0.0456) and to diazepam-treated Ts65Dn mice (*P* = 0.0237; Fig. 2c). These findings show that diazepam has opposite effects on spectral power within the 12–15 Hz and 15–30 Hz frequency ranges in control mice, but no measurable effects in Ts65Dn mice.

### Diazepam-induced changes in neural synchrony are significantly different between control and Ts65Dn mice

**Figure 3:**
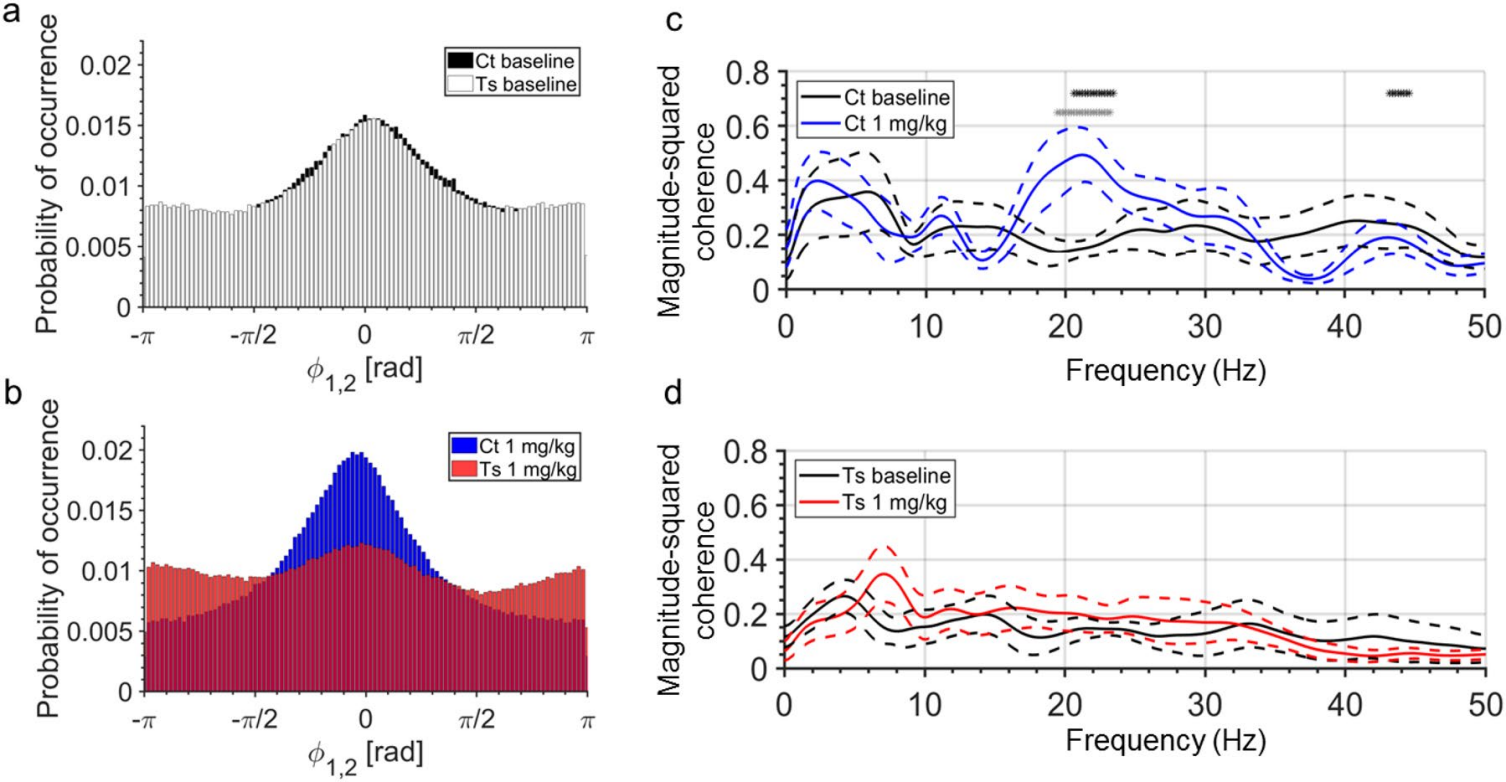
Diazepam-treated Ts65Dn mice show genotype-dependent differences in neural synchrony. (**a**, **b**) Histograms of phase differences between cortical and hippocampal EEG recordings, which were computed by dividing the phase-spectrum (0–360°) into equal-sized 100 bins (3.6°/bin). (**a**) Control and Ts65Dn mice showed similar phase-lag distributions during baseline recordings. (**b**) Diazepam at 1-mg/kg dose narrowed the peak of phase-difference distribution in control mice as the number of phase-lag estimates close to zero increased, whereas phase lag distribution of diazepam-treated Ts65Dn mice exhibited an almost uniform distribution. (**c**) Diazepam-treated control mice showed an increase in the pairwise coherence in the beta-2 frequency range (19.40–23.20 Hz; grey asterisks) compared to their pre-injection baseline. (**d**) Diazepam-treated Ts65Dn mice showed no significant changes in coherent activity compared to their pre-injection baseline. Significant differences in pairwise coherence at 20.60–23.40 Hz and 43.20–44.60 Hz frequency ranges were detected between diazepam-treated control and Ts65Dn mice (black asterisks in **c**). Number of mice: Ct baseline (N = 6), Ct 1 mg/kg diazepam (N = 6), Ts baseline (N = 7), and Ts 1 mg/kg diazepam (N = 7).

Phase synchronization plays an important role in supporting neural communication and plasticity either within the same or across brain regions^29^. To determine whether diazepam affects phase synchronization between neural oscillations, we calculated the instantaneous phase of cortical and hippocampal EEG recordings through Hilbert transform, and then we estimated the distribution of phase-difference between these pairs of signals across 0.5–50 Hz frequency range. We found that control and Ts65Dn mice showed similar phase-lag distributions during baseline recordings (*P* = 0.052, KS test with Bonferroni-adjusted P-value; Fig. 3a), with phase differences clustered around zero in both genotypes. In contrast, 1 mg/kg diazepam changed probability density functions of the phase difference between cortical and hippocampal EEG recordings in a genotype-dependent manner. Whereas the peak of phase-difference distribution became narrower as the number of phase-lag estimates close to zero increased in diazepam-treated control mice, phase lag distributions occurred with approximately equal probability in diazepam-treated Ts65Dn mice (*P* < 0.0001, KS test with Bonferroni-adjusted P-value; Fig. 3b).

We next estimated spectral coherence to measure the extent to which diazepam affects oscillatory coupling between cortical and hippocampal signals and to determine how coherent activity is distributed across frequency bins. Pairwise coherence in the beta-2 frequency range (19.40–23.20 Hz) was significantly increased by 1 mg/kg diazepam in control mice (Fig. 3c), whereas diazepam-treated Ts65Dn mice showed no significant changes in coherent activity compared to their pre-injection baseline (Fig. 3d). Significant differences in pairwise coherence at 20.60–23.40 Hz and 43.20–44.60 Hz frequency ranges were detected between diazepam-treated control and Ts65Dn mice (black asterisks; Fig. 3c). These findings show that cortical (and hippocampal) beta-2 frequency oscillations display a preferred phase-difference of coupling in diazepam-treated control mice, but not in Ts65Dn mice.

### Ts65Dn and control mice show similar susceptibility to picrotoxin-induced seizures

**Figure 4:**
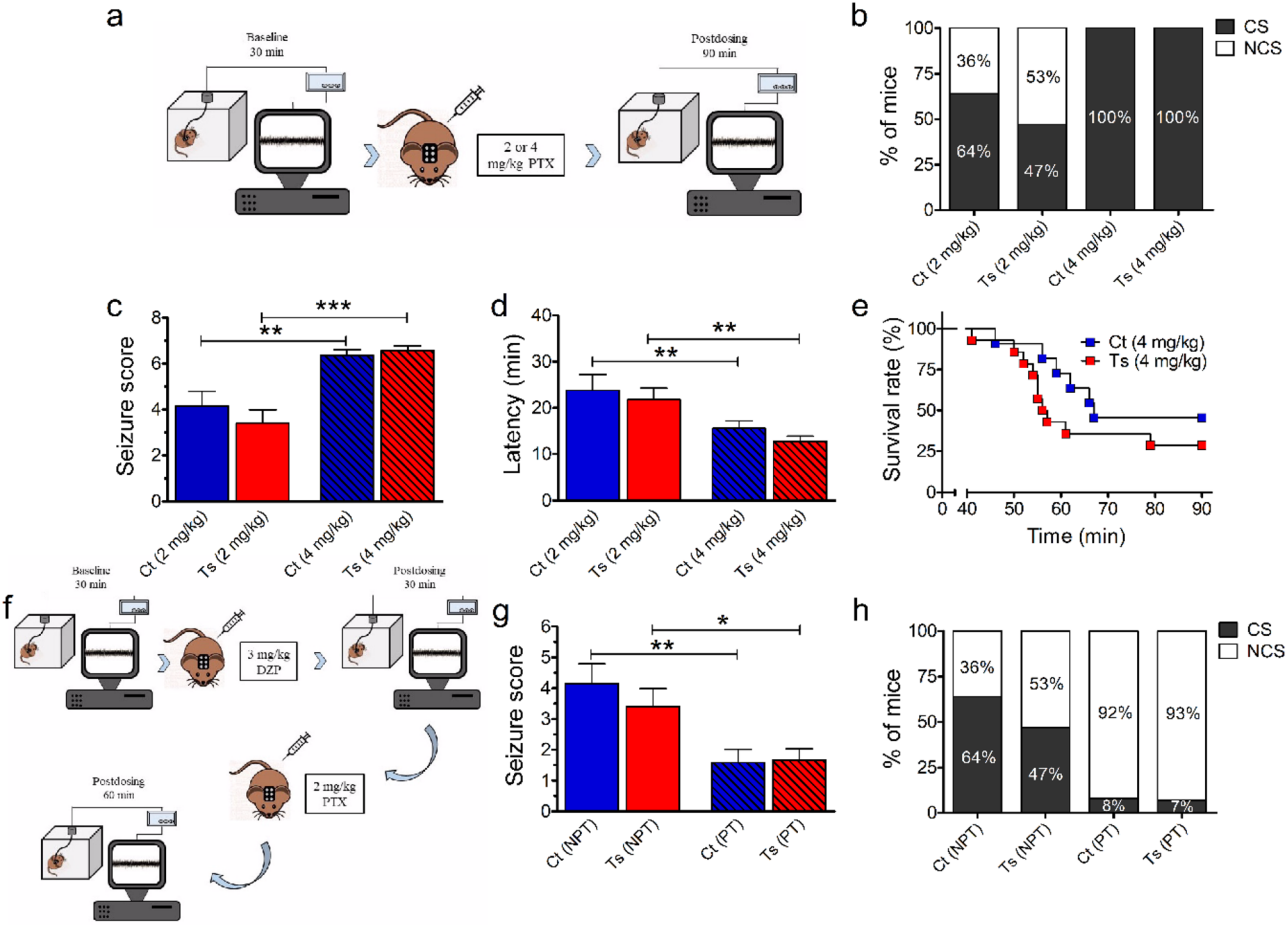
Ts65Dn and control mice display a similar dose-dependent susceptibility to picrotoxin-induced seizures, which can be effectively prevented by diazepam. (**a**) Video-EEG was recorded from control and Ts65Dn mice that were i.p. injected with picrotoxin at 2- (control, N = 14; Ts65Dn, N = 15) and 4-mg/kg doses (control, N = 11; Ts65Dn, N = 14). The experiment consisted of a 30-min baseline recording, followed by a 90-min post-dosing recording. (**b**) The administration of 2 mg/kg picrotoxin evoked convulsive seizures (CS) in approximately 64% and 47% of control and Ts65Dn mice, respectively. All mice of both genotypes exhibited CS when treated with 4 mg/kg picrotoxin. (**c**, **d**) Picrotoxin produced a dose-dependent effect on seizure severity score (**c**) and latency for CS onset (**d**) in both control and Ts65Dn mice, the highest dosage of picrotoxin (4 mg/kg) induced more severe seizures within a shorter period of time. (**e**) Survival curves for control and Ts65Dn mice treated with 4 mg/kg picrotoxin were not significantly different. (**f**) We then acutely pretreated control and Ts65Dn mice with 3 mg/kg diazepam (i.p.) before seizure-induction by 2 mg/kg picrotoxin. The experiment consisted of a 30-min baseline recording, followed by a 30-min post-dosing recording immediately after mice received an i.p. injection of 3 mg/kg diazepam. Mice were then injected with 2 mg/kg picrotoxin (i.p.) and recordings continued for an additional 60 min. (**g**) Both control and Ts65Dn pretreated with 3 mg/kg diazepam (PT) showed a decrease in the severity of picrotoxin-induced seizures compared with their respective non-pretreated peers (NPT). (**h**) Diazepam at 3-mg/kg dose almost completely prevented the occurrence of CS in both control and Ts65Dn mice (only one PT mice of each genotype developed CS). Number of mice: Ct 2 mg/kg PTX (N = 14), Ct 4 mg/kg PTX (N = 11), Ct non-pretreated with 3 mg/kg diazepam (NPT; N = 14), Ct pretreated with 3 mg/kg diazepam (PT; N = 12), Ts 2 mg/kg PTX (N = 15), Ts 4 mg/kg PTX (N = 14), Ts non-pretreated with 3 mg/kg diazepam (NPT; N = 15), and Ts pretreated with 3 mg/kg diazepam (PT; N = 15). Data represent mean ± SEM. Statistical significance is expressed as *, ** and *** for *P* < 0.05, *P* < 0.01, and *P* < 0.001, respectively.

Given that our previous findings did not produce any evidence of observable seizures or epileptiform activity induced by GABA_A_R PAM in Ts65Dn mice, we tested the effects of the inhibition of GABAergic transmission by the GABA_A_R antagonist picrotoxin on the seizure susceptibility of Ts65Dn mice. For this set of experiments, video-only or video-EEG was recorded from control and Ts65Dn mice that were intraperitoneally (i.p.) injected with picrotoxin at 2- (control, N = 14; Ts65Dn, N = 15) and 4-mg/kg doses (control, N = 11; Ts65Dn, N = 14; Fig. 4a). Then, seizure severity was scored using a modified Racine scale system^30^, in which higher values correspond to more severe seizures and a score of seven indicates death. Only fully induced convulsive (motor) seizures (classes 4–7) were considered to estimate the latency, which was defined as the time in minutes from the administration of picrotoxin to the onset of convulsive seizures.

The administration of 2 mg/kg picrotoxin evoked convulsive seizures in approximately 64% and 47% of control and Ts65Dn mice, respectively (Fig. 4b). In contrast, all animals of both genotypes exhibited convulsive seizures when treated with 4 mg/kg picrotoxin (Fig. 4b). The administration of picrotoxin also produced a dose-dependent effect on the mean values of seizure severity score as revealed by two-way ANOVA (F_(1, 50)_ = 30.002, *P* < 0.0001; Supplementary Table S11). Post hoc tests identified a significant increase in the seizure severity score for both control (*P* = 0.0035) and Ts65Dn (*P* < 0.0001) mice treated with 4 mg/kg picrotoxin compared with their 2 mg/kg picrotoxin-treated peers (Fig. 4c; Supplementary Table S12). Similarly, the mean values of latency were significantly affected by the picrotoxin dosage (F_(1, 37)_ = 16.275, *P* = 0.0003; Supplementary Table S11), which was confirmed by post hoc comparisons for the latency to convulsive seizure onset in control (*P* = 0.0089) and Ts65Dn (*P* = 0.0056) mice treated with 4 mg/kg picrotoxin compared with their 2 mg/kg picrotoxin-treated peers (Fig. 4d; Supplementary Table S12). We found that 71% of Ts65Dn mice treated with 4 mg/kg picrotoxin died during the course of the experiment, in contrast to 55% mortality in controls (Fig. 4e). Such difference, however, was not statistically significant (*P* = 0.2325, Mantel-Cox log-rank test).

### Diazepam was efficacious in preventing picrotoxin-induced seizures in both Ts65Dn and control mice

To investigate a potential genotype dependence of diazepam’s protective effect against picrotoxin-induced seizures, we administered 2 mg/kg picrotoxin i.p. to control (N = 12) and Ts6Dn (N = 15) mice pretreated with 3 mg/kg of diazepam (30 min before the picrotoxin injections). Mouse behavior was monitored for 60 min following picrotoxin injection for seizure severity scoring (Fig. 4f). The anticonvulsant activity of diazepam was considered effective when it prevented the occurrence of convulsive seizures (classes 4–7). Treatment (F_(1, 52)_ = 16.668, *P* = 0.0002), but not genotype (F_(1, 52)_ = 0.393, *P* = 0.5333) had a significant effect on the mean values of seizure severity score (Supplementary Table S11). Post hoc analysis confirmed that the acute pretreatment with 3 mg/kg diazepam significantly decreased the severity of seizures induced by the administration of 2 mg/kg picrotoxin in both control (*P* = 0.0016) and Ts65Dn mice (*P* = 0.0189) compared to the animals that did not receive the pretreatment (Fig. 4g; Supplementary Table S12). The incidence of picrotoxin-induced seizures with scores 4–7 in control and Ts65Dn mice was almost completely abolished by pretreatment with 3 mg/kg diazepam (only one pretreated mouse of each genotype developed convulsive seizures; Fig. 4h). These findings indicated that diazepam was similarly effective in reducing picrotoxin-induced seizure severity and susceptibility to convulsive seizures in both control and Ts65Dn mice.

### GABA and diazepam decrease hippocampal CA1 LTP induced by theta-burst stimulation (TBS) in control-but not in Ts65Dn-derived slices

**Figure 5:**
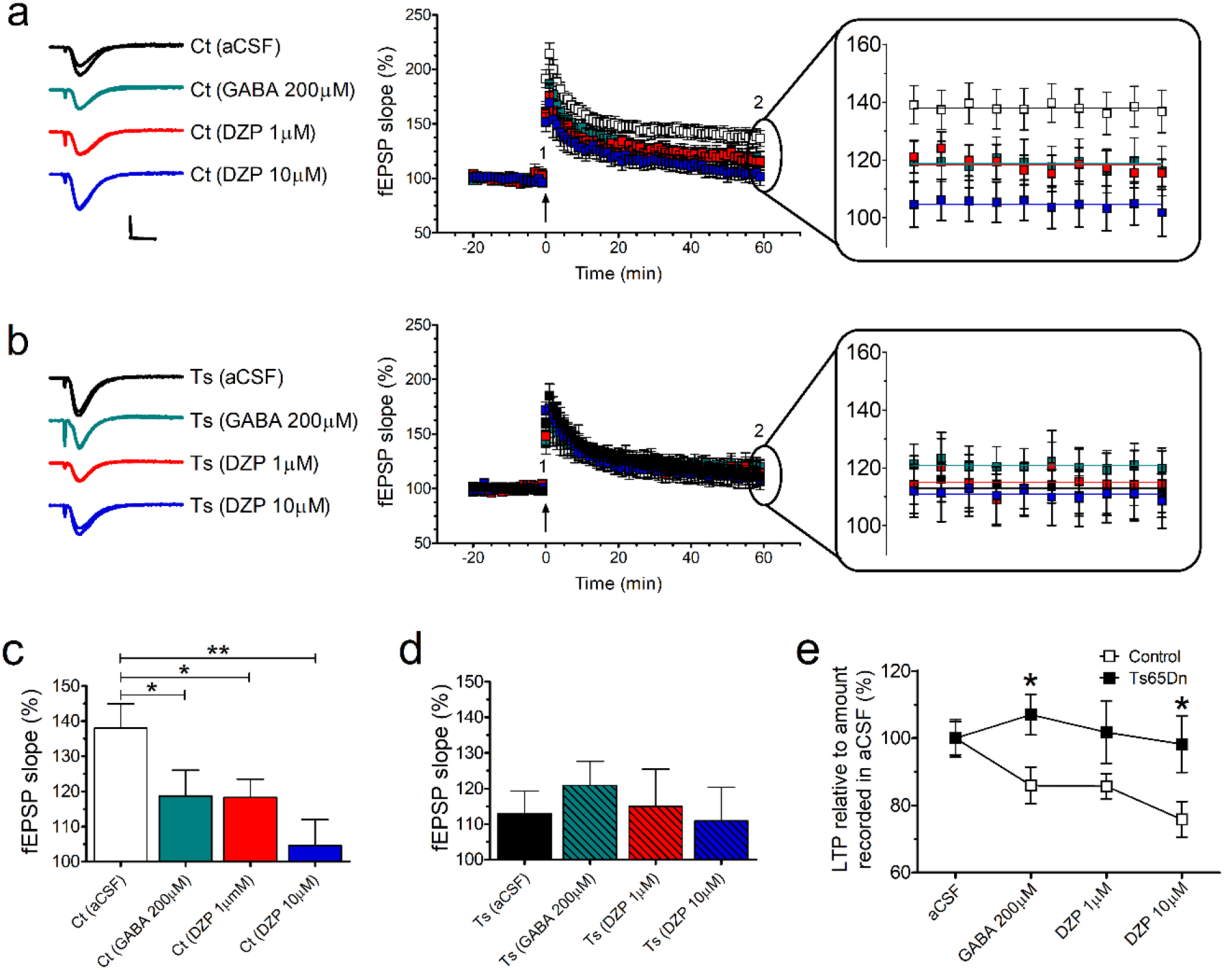
The magnitude of CA1 TBS-induced LTP is depressed by GABA and diazepam in control- but not in Ts65Dn-derived slices. (**a**, **b**) The calculated mean fEPSP slopes of TBS-induced LTP for control- (**a**) and Ts65Dn- (**b**) derived slices. Arrow indicates LTP induction, and representative traces show synaptic response during baseline (time 1) and at end of recording (time 2). The magnification of the last 10 min of recording is also shown. Scale bars represent 1 mV (horizontal) and 10 ms (vertical). (**c**, **d**) Summary graph of the LTP data from control- (**c**) and Ts65D- (**d**) derived slices (mean fEPSP slope during the last 10 min of recording). Both GABA and diazepam reduced the mean level of TBS-induced LTP in control-derived slices, whereas neither GABA nor diazepam affected the mean level of TBS-induced LTP in Ts65Dn-derived slices. (**e**) Normalized data show a greater effect of inhibition on LTP in control slices at 200 μM GABA and 10 μM diazepam than in those derived from 48 Ts65Dn mice. Number of slices (one slice per mouse for each condition): Ct aCSF (N = 12), Ct 200 μM GABA (N = 12), Ct 1 μM diazepam (N = 12), Ct 10 μM diazepam (N = 12), Ts aCSF (N = 12), Ts 200 μM GABA (N = 12), Ts 1 μM diazepam (N = 12), and Ts 10 μM diazepam (N = 12). Data represent mean ± SEM. Statistical significance is expressed as * and ** for *P *< 0.05 and *P *< 0.01, respectively.

Because the induction of hippocampal CA1 LTP is also sensitive to GABAergic inhibition^31,32^, we tested whether GABA (200 μM) or diazepam (1 and 10 μM) would modulate the magnitude of CA1 TBS-induced LTP in Ts65Dn mice. We found that both GABA and diazepam (at 1 and 10 μM) significantly decreased the mean levels of LTP in control-derived hippocampal slices (F_(3, 44)_ = 4.103, *P* = 0.0119) (Fig. 5a,c), but not in Ts65Dn-derived hippocampal slices (F_(3, 44)_ = 0.857, *P* = 0.8570) (Fig. 5b,d; Supplementary Table S6). Fisher’s LSD post hoc analysis confirmed that 200 μM GABA (*P* = 0.0489), 1 μM diazepam (*P* = 0.0458), and 10 μM diazepam (*P* = 0.0011) significantly decreased the mean levels of LTP in control-derived hippocampal slices (Fig. 5c; Supplementary Table S7).

We then normalized data to untreated genotype-specific levels as an alternative way to investigate genotype-dependent effects of GABA or diazepam on TBS-induced LTP in Ts65Dn and control mice. Two-way ANOVA revealed that genotype (F_(1, 88)_ = 10.987, *P* = 0.0013), but not treatment (F_(3, 88)_ = 1.494, *P* = 0.2217), had significant effects on the mean levels of LTP. No significant interaction was found between genotype and treatment (F_(3, 88)_ = 1.314, *P* = 0.2750; Supplementary Table S8). Post hoc analysis confirmed that control-derived hippocampal slices showed relatively greater inhibition to LTP by 200 μM GABA (*P* = 0.0211) and diazepam at 10 μM (*P* = 0.0145) compared with Ts65Dn-derived hippocampal slices (Fig. 5e; Supplementary Table S9). These findings show that TBS-induced LTP is not sensitive to GABAergic modulation by either GABA or diazepam in Ts65Dn-derived hippocampal slices.

### Ts65Dn and control mice show differential sensitivity to diazepam modulation of anxiety-like behavioral responses

**Figure 6:**
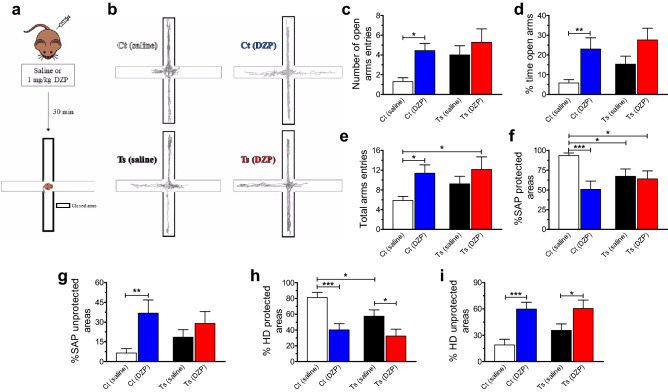
Ts65Dn mice show altered sensitivity to diazepam modulation of anxiety-like behavioral responses compared to control euploid mice. (**a**) Control and Ts65Dn mice were i.p. injected with either diazepam at 1-mg/kg dose (control, N = 16; Ts65Dn, N = 14) or saline (control, N = 16; Ts65Dn, N = 14) 30 min before testing in the EPM. (**b**) Representative trial trajectories of saline- and diazepam-treated mice exposed to EPM task. (**c**, **d**) Diazepam-treated control mice showed an increase in both the number of open arms entries (**c**) and the percentage of time spent in open arms (**d**) than vehicle-treated control mice. (**e**) Diazepam-treated control mice also showed an increase in total arms entries than vehicle-treated control mice. Regarding ethological parameters, (**f**) diazepam at 1-mg/kg dose decreased the percentage of stretched attend posture (SAP) in control mice compared with vehicle-treated control mice. Vehicle-treated Ts65Dn mice had significantly less protected SAP than vehicle-treated control mice. (**g**) Diazepam-treated control mice showed an increase in the percentage of unprotected SAP in control mice than vehicle-treated control mice. (**h**) Diazepam at 1-mg/kg dose also decreased the percentage of head-dipping (HD) in both control and Ts65Dn mice compared with their respective vehicle-treated peers. Vehicle-treated Ts65Dn mice had lesser protected HD than vehicle-treated control mice. (**i**) Both control and Ts65Dn mice treated with 1 mg/kg diazepam showed an increase in the percentage of unprotected HD compared with their respective vehicle-treated peers. Number of mice: Ct baseline (N = 16), Ct 1 mg/kg diazepam (N = 16), Ts baseline (N = 14), and Ts 1 mg/kg diazepam (N = 14). Data represent mean ± SEM. Statistical significance is expressed as *, ** and *** for *P* < 0.05, *P* < 0.01, and *P* < 0.001, respectively.

We used an EPM behavioral protocol to address how acute treatment with diazepam modulates anxiety-like responses in Ts65Dn mouse model. Control and Ts65Dn mice (8–12 weeks old) received either diazepam (control, N = 16; Ts65Dn, N = 14) at an anxiolytic but not sedative dose (1 mg/kg^33^) or vehicle (saline; control, N = 16; Ts65Dn, N = 14) 30 min before testing in the EPM (Fig. 6a). Then, both standard parameters of spatiotemporal distribution patterns of exploratory activity across the maze (Fig. 6b) and ethological measures (which are used to assess a range of specific behaviors related to the murine defensive repertoire) were determined.

We found that treatment with diazepam (F_(1, 56)_ = 6.123, *P* = 0.0164), but not genotype (F_(1, 56)_ = 3.935, *P* = 0.0522), had significant effects on the mean values of open arm entries. No significant interaction was found between genotype and treatment (F_(1, 56)_ = 1.065, *P* = 0.3066; Supplementary Table S13). Post hoc test revealed that diazepam-treated control mice entered open arms more frequently than vehicle-treated control mice (*P* = 0.0130; Fig. 6c; Supplementary Table S14). Similar results were observed for the analysis of percentage of time spent in open arms, which resulted in significant dependence on treatment (F_(1, 56)_ = 10.124, *P* = 0.0024), but not on genotype (F_(1, 56)_ = 2.337, *P* = 0.1319). Again, no significant interaction between genotype and treatment was found (F_(1, 56)_ = 0.290, *P* = 0.5921; Supplementary Table S13). Post hoc tests showed that the percentage of time spent in open arms was significantly higher for diazepam-treated control mice than for vehicle-treated control mice (*P* = 0.0086; Fig. 6d; Supplementary Table S14). Finally, analysis of total arms entries showed a significant dependence on treatment (F_(1, 56)_ = 5.947, *P* = 0.0179), but not on genotype (F_(1, 56)_ = 1.412, *P* = 0.2397). No significant interaction was found between genotype and treatment (F_(1, 56)_ = 0.553, *P* = 0.4600; Supplementary Table S13). Post hoc comparisons showed that total arms entries were almost two times as high for diazepam-treated control mice as for vehicle-treated control mice (*P* = 0.0235; Fig. 6e; Supplementary Table S14). Data on treatment effects on the mean values of closed arm entries can be found in Supplementary Fig. S5 and Supplementary Table S13. These findings show that diazepam abolished the preference shown by saline-treated control mice for protected areas of the EPM, which is supported by the significant increase in the number of open arm entries and time spent in open arms without affecting the number of closed arm entries and time spent in closed arms. However, Ts65Dn mice treated with diazepam did not differentiate between areas of the EPM.

We then scored ethological patterns of response as a means to assess behavioral strategies (i.e. risk assessment behaviors) expressed by mice when dealing with stressful situations. First, we assessed the frequency of stretched attend posture (SAP), which is characterized by a forward elongation of the animal’s body followed by a retraction to its original position. SAP was differentiated as protected (i.e., occurring on/from the closed arms or central platform) or unprotected (occurring on/from the open arms). Two-way ANOVA showed that treatment (F_(1, 56)_ = 6.955, *P* = 0.0108), but not genotype (F_(1, 56)_ = 0.551, *P* = 0.4611), had significant effects on the percentage of protected SAP [(protected/total) × 100]. A significant interaction was found between genotype and treatment (F_(1, 56)_ = 5.104, *P* = 0.0278; Supplementary Table S13). Post hoc tests revealed that vehicle-treated Ts65Dn mice had significantly less protected SAP than vehicle-treated control mice (*P* = 0.0383; Fig. 6e). Treatment with diazepam significantly decreased protected SAP in control mice when compared to vehicle-treated control mice (*P* = 0.0007; Fig. 6e; Supplementary Table S14). Regarding the percentage of unprotected SAP [(unprotected/total) × 100], analysis showed that treatment (F_(1, 56)_ = 7.253, *P* = 0.0093), but not genotype (F_(1, 56)_ = 0.071, *P* = 0.7903), had significant effects on this parameter. No significant interaction was found between genotype and treatment (F_(1, 56)_ = 1.721, *P* = 0.1949; Supplementary Table S13). Post hoc tests showed that the percentage of unprotected SAP was significantly higher for diazepam-treated control mice than for vehicle-treated control mice (*P* = 0.0049; Fig. 6f; Supplementary Table S14).

Next, we assessed the frequency of head-dipping (HD), which consists of an exploratory movement wherein the mouse projects its head/shoulders over the sides of the open arms and down towards the floor. As SAP, HD was also differentiated as protected or unprotected. Both genotype (F_(1, 56)_ = 4.121, *P* = 0.0471) and treatment (F_(1, 56)_ = 18.087, *P* = 0.0001) had significant effects on the percentage of protected HD [(protected/total) × 100]. No significant interaction was found between genotype and treatment (F_(1, 56)_ = 1.058, *P* = 0.3082; Supplementary Table S13). Post hoc tests confirmed that vehicle-treated control mice performed protected HD most frequently than vehicle-treated Ts65Dn mice (*P* = 0.0349) and diazepam-treated control mice (*P* = 0.0003; Fig. 6g). Vehicle-treated Ts65Dn mice performed protected HD most frequently than diazepam-treated Ts65Dn mice (*P* = 0.0314; Fig. 6g; Supplementary Table S14). Regarding the percentage of unprotected HD [(unprotected/total) × 100], treatment (F_(1, 56)_ = 17.866, *P* = 0.0001), but not genotype (F_(1, 56)_ = 1.214, *P* = 0.2752), had significant effects on this parameter. No significant interaction was found between genotype and treatment (F_(1, 56)_ = 1.045, *P* = 0.3111; Supplementary Table S13). Post hoc tests showed that the percentage of unprotected HD was significantly higher for diazepam-treated control (*P* = 0.0003) and Ts65Dn mice (*P* = 0.0324; Fig. 6h; Supplementary Table S14) than for vehicle-treated mice of the same genotype. These findings indicated that diazepam-treated control and Ts65Dn mice exhibited a similar pattern of exploratory behavior, but vehicle-treated Ts65Dn mice appeared “less fearful” or “distressed” than vehicle-treated control mice when exposed to EPM. (Analyses on frequency of closed arm returns and grooming time can be found in Supplementary Fig. S5 and Supplementary Tables S13 and S14).

## Discussion

In this study, we investigated the effects of diazepam on brain activity, synaptic plasticity, and behavior in Ts65Dn and control euploid mice and found distinct, genotype-dependent drug responses. When compared to control animals, we found that diazepam neither increased beta-2 activity nor decreased the power of gamma and beta-1 oscillations in Ts65Dn mice. Although we observed slowing effects of diazepam on delta oscillations in both control and Ts65Dn mice, a similar effect on frequencies within the theta range was only found in control mice. Our findings also revealed that diazepam modulated the dynamics of phase relationships between brain areas in a genotype-dependent manner, with increased phase synchronization between oscillatory activities in control mice and opposite effects in Ts65Dn mice. In a separate set of experiments, we found that diazepam was effective in reducing seizure susceptibility and severity to picrotoxin-induced convulsive seizures in both control and Ts65Dn mice. Differences in diazepam effects were observed in the magnitude of CA1 TBS-induced LTP, which was depressed by diazepam in control- but not in Ts65Dn-derived slices. Finally, we observed genotype-dependent differences in sensitivity to the modulation of anxiety-like responses by diazepam. Together, these findings highlight the complex interplay between the modulation of GABA_A_R-mediated signaling in electrophysiological and behavioral outcomes in Ts65Dn mice.

The finding that control mice treated with 1 mg/kg diazepam showed a 30% increase in beta-2 activity compared to its EEG baseline recording is consistent with previous findings from several preclinical and clinical studies^23–26,34–36^. However, no evidence of diazepam-induced increase in EEG beta-band power was found in Ts65Dn mice. The specific reasons for this genotype-dependent difference are yet to be determined, but we know that the beta rhythm is closely tied to the coupling of excitatory and inhibitory cells in cortical tissue^37^. Given previous evidence on abnormalities in synaptic structure and function in Ts65Dn mice, we speculate that the beta-related electrophysiological phenotype displayed by diazepam-treated Ts65Dn mice may, at least in part, reflect abnormalities in reciprocally connected networks of inhibitory GABAergic interneurons in cortical areas of Ts65Dn mice.

The generation of gamma oscillations depends on neural synchronization either by mutual inhibition between interneurons or by the excitatory-inhibitory loop^38^. In both models, inhibition is mediated by GABA_A_R^38^. Previous studies showed that diazepam appears to modulate both the amplitude and frequency of gamma oscillations^39^, but not to disrupt their synchrony or rhythmicity^40^. Given that the modulation of GABA_A_R-mediated synaptic inhibition can control gamma-band rhythmogenesis^38,39^, we hypothesize that the lack of diazepam effect on the modulation of gamma/beta-1 activity may be associated with an impairment of such rhythmicity control mechanisms in Ts65Dn mice. This is consistent with recent findings reporting that spontaneous network oscillations (UP states) were significantly shorter and less frequent in the neocortex of Ts65Dn mice^41^. The dynamics of these spontaneous patterns of massive, persistent network activity reflect a complex, stereotypically organized sequential spread of activation through local cortical networks^42^ in which fast-spiking GABAergic interneurons expressing parvalbumin (PV^+^ cells) and deep pyramidal cells play a critical role^43–45^. The density of PV^+^ cells across multiple cortical layers was found to be increased in Ts65Dn mice by some investigators^46,47^. However, Zorrilla de San Martin and collaborators^48^ reported that perisomatic inhibition from PV^+^ interneurons was unaltered in Ts65Dn mice. Instead, these investigators observed that these cells lost their classical fast-spiking phenotype and exhibited increased excitability. In either one of these scenarios, it is possible that an altered pattern of organization of excitatory and inhibitory cell assemblies in Ts65Dn vs. control euploid mice may account for the genotype-dependent differences in cortical network dynamics observed after diazepam treatment.

Disruption of excitation and inhibition balance (E/I balance), which is critically involved in the stability of network dynamics^38^, has been reported in Ts65Dn mice and inferred in persons with DS^49^. Recent findings showing a delayed GABA polarity switch in the hippocampus of neonatal Ts65Dn mice suggest developmental delays in maturation of neural circuits of these animals, which in turn may contribute to the disruption of E/I balance towards inhibition in adult Ts65Dn mice^50^. In addition, Zorrilla de San Martin et al.^48^ reported that the dendritic inhibitory loop involving excitatory pyramidal neurons and Martinotti cells (which are somatostatin-positive interneurons that inhibit distal dendrites of excitatory pyramidal neurons) is enhanced in Ts65Dn mice^48^. Since α5-containing GABA_A_R mediate synaptic inhibition of pyramidal cell dendrites by Martinotti cells^51^, these findings may support previous evidence on the rescue of learning/memory deficits and synaptic plasticity deficits in Ts65Dn mice by negative allosteric modulators (NAMs) of these receptors^52–55^. Our findings also revealed that diazepam differently modulates phase relationships between brain areas in control and Ts65Dn mice. We found that diazepam-treated control mice showed a narrower distribution of phase differences when compared to diazepam-treated Ts65Dn mice, which suggests that diazepam increased phase synchronization between oscillatory activities of cortex and hippocampus in control mice, while it had an opposite effect in trisomic animals. We also observed a greater consistency of the phase difference (i.e., a higher coherence) between cortical and hippocampal oscillations in the beta-2 frequency range of diazepam-treated control mice compared to diazepam-treated Ts65Dn mice.

Brain oscillations at different frequencies can interact with each other via cross-frequency coupling^56,57^. Previous findings showed that, although diazepam changed the frequencies in which cross-frequency coupling occurs due to its slowing effect on frequencies, the strength of phase-amplitude coupling between slow and fast oscillations was unaffected by the drug^58^. Although we did not directly assess the properties of the phase–amplitude coupling between theta and gamma oscillations, we did see modulatory effects of 1 mg/kg diazepam on theta frequencies and gamma power in control mice, but not in Ts65Dn mice. We thus speculate that the GABA_A_R-mediated mechanisms underlying the coordination of oscillatory activity across cell assemblies are impaired in Ts65Dn mice brain, which is consistent with recent evidence suggesting abnormal prefrontal–hippocampal functional connectivity across different brain states in Ts65Dn mice^59^.

The administration of picrotoxin induced a dose-dependent increase in seizure severity and a decrease in latency to convulsive seizure onset in both control and Ts65Dn mice. Acute pretreatment with 3 mg/kg diazepam, however, significantly decreased seizure severity as well as the incidence of seizures with scores 4–7 similarly in mice of both genotypes, which demonstrates that diazepam was typically effective in reducing Ts65Dn mice susceptibility to picrotoxin-induced seizures.

The strength of GABAergic transmission has been shown to modulate plasticity at excitatory synapses on principal cells, which is a key mechanism underlying learning and memory encoding. We and others had previously shown that reduced levels of LTP in the hippocampus of Ts65Dn mice can be pharmacologically enhanced by the antagonism of GABA_A_-mediated synaptic inhibition with picrotoxin^11–14^. Here, we found that diazepam produced a significant depression in the mean level of TBS-induced LTP in control- but not in Ts65Dn-derived slices at 1 and 10 μM. Similar results were observed when control- and Ts65Dn-derived hippocampal slices were treated with 200 μM GABA. Together, these results extend previous findings on the role of GABA_A_-R in neuroplasticity dysfunction observed in Ts65Dn mice. In fact, they underscore the high-level of complexity of GABA_A_-mediated signaling in such animals since GABA_A_R appears sensitive to inhibition by picrotoxin, but less sensitive to diazepam-mediated modulation. Diazepam acts specifically through GABA_A_R containing α1, α2, α3 or α5, but not α4 or α6 subunit types, whereas picrotoxin exerts a noncompetitive inhibition via any combination of subunit subtypes. Therefore, the pattern of subunit arrangement of GABA_A_R, as well as their synaptic localization, might account for the pharmacological genotype-dependent differences in LTP levels displayed by control- and Ts65Dn-derived slices when treated with picrotoxin and diazepam. This is especially interesting in light of findings that NAMs of α5-containing GABA_A_R can enhance induction of LTP in Ts65Dn mice, as well as improve mouse behavioral performance without causing anxiogenic side effects or seizures^52–55^.

Given the crucial role of GABAergic function in modulating anxiety responses, an additional goal of this study was to investigate both anxiety-related behavioral differences between Ts65Dn and control mice in EPM exploration and the effects of diazepam treatment. To this end, both standard and ethological parameters were considered. In the first set of analyses, similar spatiotemporal patterns of exploratory activity were found between vehicle-treated groups. Both Ts65Dn and control mice preferred closed over open arms, which is consistent with the natural tendency of mice to avoid open areas in favor of enclosed spaces^60^. The ethological approach, however, allowed us to detect significant differences between defensive elements displayed by trisomic and euploid mice during EPM testing. For instance, Ts65Dn mice exhibited a relatively lower percentage of protected movements of SAP and HD. In addition, trisomic mice turned back into (rather than leaving) a closed arm less often than controls. Together, these results suggest that the differences observed in behavioral responses of Ts65Dn mice may stem from a lower level of anxiety when faced with approach-avoidance conflicts compared to the control mice. Alternatively, these behavioral responses of Ts65Dn mice may simply reflect a failure of this animal in recognizing, attending to, or interpreting environmental cues appropriately^61^. It is also possible that Ts65Dn mice have a decreased ability to recognize the aversiveness represented by the environmental context of the EPM, which may reflect deficits in executive function^62–65^.

Genotype-dependent differences in sensitivity to the anxiolytic properties of diazepam were also detected by EPM testing. Diazepam produced a significant increase in both time spent and number of entries into the open arms in control mice, while it had no major effects on spatiotemporal patterns of exploratory activity in Ts65Dn mice. We also observed that diazepam reduced risk assessment behaviors in both control and Ts65Dn mice. Considering the absence of a significant diazepam effect on percentage time and number of open arm entries in Ts65Dn mice, and the fact that risk assessment behaviors are very sensitive to anxiolytic drugs^66^, our findings suggest that diazepam may have induced a more subtle anxiolytic effect in Ts65Dn mice compared to control mice.

We cannot ignore the potential for genotype-dependent differences in pharmacokinetics and/or pharmacodynamics of diazepam effects. For instance, given diazepam’s high lipophilicity^67^, genotype-dependent differences in body composition may also affect drug distribution and rate of blood–brain barrier penetration. It is also possible that regional differences in GABA_A_R subunit composition between Ts65Dn and control mice may contribute to the differential profile of responses displayed by animals of these two genotypes.

Many questions remain about the precise mechanisms behind the genotype-dependent differences in responses to diazepam documented in the present work, but it is clear that our findings do not support a global reversal of GABA_A_R responses from inhibitory to excitatory in the Ts65Dn mouse model of DS. Not only diazepam did not produce seizures in Ts65Dn mice, but, in agreement with clinical observations in patients with DS, it was similarly effective in reducing susceptibility and severity of picrotoxin-induced convulsive seizures in Ts65Dn and control euploid mice. Although it is still possible that Cl^−^ homeostasis may be altered in limited brain regions of Ts65Dn mice, we should note that none the results of the present work, including in vitro work on hippocampal slices, support this notion.

The dysfunctional GABA_A_R-mediated neurotransmission phenotypes reported here should prove useful in enhancing our understanding of some of the neural substrates for the cognitive deficits observed in persons with DS. Furthermore, these findings also provide preclinical support for the use of EEG techniques for monitoring therapeutic responses in patients with DS. For example, the validation of specific EEG biomarkers of brain functioning may prove helpful in discriminating between potentially effective and dead-end pathways on the road toward improved clinical care of patients with DS with or without neurological and/or psychiatric co-morbidities.

## Methods

### Mice

All experimental procedures were approved by the Institutional Animal Care and Use Committee (IACUC) at Case Western Reserve University (CWRU) and were carried out in compliance with the ARRIVE guidelines and in accordance with the Guide for the Care and Use of Laboratory Animals (National Institutes of Health, 2011). Ts65Dn mice were generated by in-house repeated backcrossing of Ts65Dn females (obtained from The Jackson Laboratory, Bar Harbor, ME) with B6EiC3Sn.BLiAF1/J hybrid male mice (C57BL/6JEiJ females x C3Sn.BLiA-*Pde6b*^+^/Dn males, also purchased from The Jackson Laboratory). The colony was maintained in a controlled 12:12 light/dark cycle (lights off at 6:00 p.m.) in the Animal Resource Center at CWRU. Animals were provided with food and water ad libitum. Ts65Dn mice were genotyped using polymerase chain reaction (PCR)^68^. Euploid littermates of Ts65Dn mice were used as control mice.

### Electrode preparation

Three bare silver wires (127 μm diameter; A-M Systems, Inc., Carlsborg, WA, USA) and two formvar-insulated nichrome wires (66 μm diameter; A-M Systems, Inc., Carlsborg, WA, USA) were soldered into separate channels of an eight-channel headstage (Plexon Inc., Dallas, TX, USA). Two silver wires were used as recording electrodes, while the other one was used as reference electrode. The bipolar electrode was made by twisting the nichrome wires around each other.

### Surgical procedure for electrode implantation

Mouse survival surgeries were performed using aseptic techniques. Control (N = 6) and Ts65Dn (N = 7; 13–20 weeks old) male mice were anesthetized with ketamine and xylazine (95 mg/kg and 5 mg/kg, respectively, as a single intraperitoneal (i.p.) injection of 6.25 ml/kg), and then placed in the stereotaxic apparatus (Model 1900 Stereotaxic Alignment System, David Kopf Instruments, Tujunga, CA, USA). Body temperature was maintained at approximately 37 °C using a thermal pad. After fixing the mouse’s head, the scalp was locally anesthetized with 2% lidocaine hydrochloride subcutaneous (s.c.) injection and the skull was exposed by a small midline incision. Hydrogen peroxide-soaked cotton swabs were used for removing any remaining connective tissue on the skull surface. Two small craniotomies (~ 0.5 mm diameter) were drilled on both sides of the frontal cortex (anterioposterior AP: + 1.5 mm, mediolateral ML: ± 1.5 mm)^69^ for screw fixation. One more craniotomy was performed above the parietal cortex for positioning the bipolar electrode into the left hippocampal CA1 area (AP: − 2.3 mm, ML: − 1.7 mm; and dorsoventral DV: − 1.2 mm)^69^. The reference screw was placed above the contralateral parietal cortex. The stainless steel screws and the exposed skull were covered by Teets 'Cold Cure' dental cement (A-M Systems, Inc., Carlsborg, WA, USA). Carprofen (5 mg/kg, s.c. injection) was used for preemptive analgesia and within 24 h following surgery. Animals were singly housed after surgery and allowed to recover at least one week before being submitted to video-EEG monitoring.

### Drug treatments

Diazepam was purchased from Hospira, Inc. (5 mg/ml; Lake Forest, IL, USA) and was diluted to 0.16, 0.48, and 1.6 mg/ml with 0.9% saline solution prior to administration. This diazepam formulation contains 40% propylene glycol, 10% alcohol, 5% sodium benzoate and benzoic acid added as buffers, and 1.5% benzyl alcohol added as a preservative. Diazepam was administered i.p. to mice in a volume of 6.25 ml/kg body weight. The dose–response relationships for diazepam in Ts65Dn mice were evaluated following single administration of 1-, 3-, and 10-mg/kg doses. The rationale for choosing these doses was based on previous findings showing that 1 mg/kg diazepam was effective in reducing anxiety-like behaviors^33^, whereas 3 mg/kg diazepam was effective against seizures^70^. Finally, 10 mg/kg diazepam is a behaviorally disruptive dose, wherein treated-animals showed gait abnormalities, hypoactivity, and reduced muscle tone^71^. For these in vivo experiments, 0.9% saline solution was used as the placebo, which obviated the use of three separate vehicle-treated groups. There were three main reasons supporting this choice of vehicle in the placebo group. First, the v/v % of saline vs. the Hospira diazepam formulation was 97, 90, and 68 for 1-, 3-, and 10-mg/kg doses, respectively, which means that saline solution was the most representative constituent of the injections (mainly at the key doses of 1- and 3-mg/kg). Second, the corresponding dose of ethanol (perhaps the most potentially problematic vehicle component) in this formulation as used here were equivalent to 0.016, 0.048, and 0.158 g/kg for 1-, 3-, and 10-mg/kg diazepam doses, respectively. This means that, even at the maximum diazepam dose, the amount of ethanol in the Hospira formulation is well below any minimum ethanol dose in the literature necessary to produce detectable behavioral effects. Third, and most important, the Hospira formulation is a veterinarian-grade drug that is identical to the diazepam injections that have been used in human beings at emergency rooms across the globe for decades, and the effects of such clinical formulations have always been attributed to diazepam and not to any of its vehicle components. Still, one cannot totally discount the very small possibility that the lack of identical chemical contents in the vehicle-treated group could have a minor effect on the interpretation of diazepam effects vis-à-vis the placebo group in this subset of experiments.

Picrotoxin was purchased from Sigma-Aldrich (St. Louis, MO, USA) and was diluted to 0.32 and 0.64 mg/ml with 0.9% saline solution prior to administration. Picrotoxin was administered i.p. to mice as individual single doses of 2 and 4 mg/kg in a volume of 6.25 ml/kg body weight. Individual mice were subjected to all experimental conditions with inter-injection intervals of at least 24 h.

### Video and video-EEG recordings

To determine the behavioral effects of diazepam on Ts65Dn (N = 15) and control (N = 14) mice (13–20 weeks old), video recordings were performed in freely moving mice acutely treated with 1, 3, or 10 mg/kg diazepam (i.p.). Subsets of Ts65Dn (N = 7) and control (N = 6) animals were previously chronically implanted with EEG electrodes to record brain electrical activity during these video recording sessions. A webcam (C920 HD Pro Webcam, Logitech, Newark, CA, USA) was placed above the recording chamber for continuous monitoring of mouse behavior. For the video-EEG subset of animals, on the day of the experiment, mouse head mount was connected to the recording apparatus (OmniPlex Neural Recording Data Acquisition System, Plexon Inc., Dallas, TX, USA) by an interface cable. Such cable was attached to a commutator, which allowed mice to move freely in the recording chamber. After 30 min of recording of baseline activity, mice were i.p. injected with one of the following: (1) 1 mg/kg diazepam; (2) 3 mg/kg diazepam; (3) 10 mg/kg diazepam; (4) 2 mg/kg picrotoxin; or (5) 4 mg/kg picrotoxin. Each injection was followed by data acquisition for a period of 90 min. To investigate the efficacy of diazepam in preventing picrotoxin-induced seizures, immediately after 30-min baseline recording, mice were injected with 3 mg/kg diazepam and recordings were continued for another 30 min. After that, mice received 2 mg/kg picrotoxin and recordings were continued for 60 min. The 2-h EEG recording sessions were carried out in the light phase of the circadian cycle.

### EEG data analysis

Local field potentials were acquired at 2-kHz sampling rate and band-pass filtered (3–500 Hz, four-pole Butterworth filter). The adaptive power line noise removal was used to remove 60 Hz and its five first harmonics. Signal processing was carried out off-line by custom-written MATLAB codes (The MathWorks Inc., Natick, MA, USA). Multiple artifact-free EEG segments within the 30 min of baseline recording (prior to diazepam administration) and within the 30-min post-dosing period were manually selected for further analysis. We specifically focused on this period based on previous findings showing that diazepam and its active metabolites achieve maximum concentration in mouse brain within 30 min after dosing^33^.

Power spectral density (PSD) was estimated by Welch's method (window-size: 5 s; window function: Hamming; overlap: 50%). The PSD was calculated for each animal of each experimental group and then a mean PSD was estimated per group. For the analysis of band power, mean frequency, power of the mean frequency, skewness, and kurtosis we partitioned the PSD into the wavebands delta (0.5–4 Hz), theta (4–8 Hz), alpha (8–12 Hz), beta 1 (12–15 Hz), beta 2 (15–30 Hz), gamma (30–50 Hz), and high gamma (50–100 Hz)^72^. Because we found no significant differences between data from left and right cortical derivations, EEG recordings from both hemispheres of a given mouse in a given experimental condition were averaged to represent that animal in further analysis.

To determine phase-difference between hippocampal and cortical electrodes, raw time series data were band-pass filtered (0.5–50 Hz), and then the instantaneous phase was estimated from the complex-valued analytic signal via Hilbert transform in each time window. Phase-difference histograms were computed by dividing the phase-spectrum (0°–360°) into equal-sized 100 bins (3.6°/bin), and the number of observed events within each bin was determined in order to quantify its empirical probability density function.

To quantify the strength of the relationship between hippocampal and cortical signals within each frequency bin, spectral coherence was determined as a function of the PSD by dividing the square of the absolute value of the cross-spectral density function of these two signals by the product of the autospectral density function of each signal^73^. The coherence ranges from 0 to 1, wherein 1 denotes maximum coherence and 0 indicates no coherence between signals. The power of each waveband was calculated as the area under the power spectrum curve within a frequency range of interest and then it was expressed as a ratio of the total power of the signal.

### Field excitatory postsynaptic potential (fEPSP) recordings

Control (N = 12) and Ts65Dn (N = 12; 3–6 months old) male mice were used in these experiments. Mice were deeply anesthetized with halothane and rapidly decapitated. Brains were then quickly dissected in ice-cold (4 °C), saturated (95% O_2_/5% CO_2_) artificial cerebral spinal fluid (aCSF) solution containing (in mM): 120 NaCl, 3.5 KCl, 2.5 CaCl_2_, 1.3 MgSO_4_, 1.25 NaH_2_PO_4_, 26 NaHCO_3_, and 10 D-glucose. Slices were cut with a vibrating blade microtome (VT 1000 s, Leica, Bannockburn, IL) into transverse, 400 μm thick slices. Hippocampal slices were dissected out and allowed to recover for at least an hour at room temperature in a holding chamber containing oxygenated aCSF. After this period, hippocampal slices were transferred to the recording chamber and superfused with aCSF, aCSF with 200 μM GABA, aCSF with 1 μM diazepam, or aCSF with 10 μM diazepam solutions at 30 ± 0.5 °C.

Stock solutions of GABA^74,75^ (Sigma-Aldrich, St. Louis, MO) were dissolved in standard aCSF at 200 mM, stored at − 80 °C until the day of experiments, and diluted 1:1000 to a final concentration of 200 μM on the day of experiments. Stock solutions of diazepam^76–78^ (Sigma-Aldrich, St. Louis, MO) was first dissolved in standard aCSF containing 0.01% DMSO at 1 mM and 10 mM, stored at − 80 °C until the day of experiments, and diluted 1:1000 to a final concentration of 1 μM and 10 μM on the day of experiments. In the final aCSF, GABA, and diazepam solutions, there was less than 0.001% DMSO.

To reduce day-to-day variations, parallel recordings from untreated (aCSF only) and treated (aCSF containing GABA or diazepam) hippocampal slices of control and Ts65Dn mice were performed using a customized electrophysiology setup as described previously^12,13^. In all experiments, one hippocampal slice was used per genotype per condition. Field excitatory postsynaptic potentials were recorded with Ag/AgCl recording electrodes through thin-walled, 1.5 mm, WPI borosilicate glass micropipettes filled with aCSF (3–5 MΩ resistance), inserted into the CA1 region of the hippocampus. Two fine bipolar platinum/iridium electrodes (FHC, Bowdoinham, ME) were positioned on the CA1 Schaffer collateral fibers at opposite sides of the recording pipette. Stimulation intensity was adjusted to 40–50% of the amplitude that is required to produce population spikes. For all experiments, a stable baseline of synaptic transmission was established for 20 min prior to the induction of LTP. LTP was then induced by theta-burst stimulation (TBS, five bursts of four pulses at 100 Hz, 200 ms intertrain interval) in the CA1 region of Ts65Dn- and control-derived hippocampal slices and the fEPSPs were recorded for an additional 60 min. Signals from the recording electrode were amplified 1000 times (Brownlee Precision Electrophysiology Amplifier Model 440, San Jose, CA), low-pass filtered (8-pole Bessel) at 2 kHz, and digitized at 20 kHz by a Digidata digitizer (1322A, Axon Instruments) into a Microsoft Windows-based computer. PCLAMP software (PCLAMP 8.2, Axon Instruments) was used for data acquisition and offline analysis. Synaptic efficacy was determined by the slope of fEPSPs normalized to the mean value of fEPSP slopes recorded prior to the LTP induction.

### Elevated plus-maze (EPM) behavioral test

Control (N = 32) and Ts65Dn (N = 28; 8–12 weeks old) male mice were injected with either vehicle (saline) or 1 mg/kg diazepam (i.p.) 30 min before being tested in the EPM. The EPM apparatus used here consisted of two sets of opposing arms (30 × 5 cm) connected by a central platform (5 × 5 cm). Two of the arms were enclosed by transparent walls (15 cm high), while the other two arms were open. The open arms had a 5 mm high border to minimize animal falls. The height of the platform was 40 cm, and it was set at the center of an empty, circular tank to protect the mouse from falling or attempting to escape during the test. Home cages were transferred to the testing room at least 1 h prior to the beginning of the test. Each mouse was placed in the central area of the maze, facing one of the open arms, and tested for 5 min. The test was recorded using a digital camera mounted above the apparatus. The ToxTrac software^79^ was used for analyzing spatiotemporal parameters, while ethological measures were scored by a trained observer blind to mouse genotype and treatment. However, regarding genotype, we know historically in our group that a trained observer can visually identify a Ts65Dn mouse from its euploid counterpart with approximately 70% accuracy. For drug treatment, one of the lab team members (typically the lab PI) prepared the study medication and labeled the vial with a randomization code. The experimenter then recorded and analyzed the video session without knowledge on whether she was analyzing behavior resulting from drug or saline injections.

### Statistical analysis

Results are presented as mean ± standard error of the mean (SEM). MATLAB (The MathWorks, Inc., Natick, MA), Statistica Academic (version 13, TIBCO Software, Palo Alto, CA), and GraphPad Prism (version 7.0, GraphPad Software, La Jolla, CA) were used for statistical analyses. One-way multivariate analysis of variance (MANOVA) was performed to compare means between genotypes (control versus Ts65Dn mice) across several dependent variables (EEG spectral power across multiple combinations of frequencies between 0.5 and 50 Hz in 2-Hz steps) at 1, 3, and 10 mg/kg diazepam. One-way MANOVA was followed by a canonical discriminant analysis to determine the ability of a set of variables (i.e., EEG spectral power across multiple combinations of frequencies) to group a sample into clusters (diazepam-treated control and Ts65Dn mice at 1-, 3-, and 10 mg/kg doses). Mean values of the first and second canonical variables (*Can1* and *Can2*, respectively) were compared between groups by one-way analysis of variance (ANOVA). Mean values of PSD were compared between baseline and diazepam conditions for each dose in each genotype by two-tailed paired t tests. For comparisons between genotypes, the mean PSD for the 30-min post-dosing EEG segments was divided by the mean PSD for the 30-min baseline-recording EEG segments in each experimental condition and then the normalized PSD for each diazepam dose was compared between genotypes by two-tailed unpaired t tests. Mean values of band power, mean frequency, power of the mean frequency, skewness, and kurtosis were compared between baseline and diazepam conditions for each dose by repeated measures analysis of variance (RM ANOVA), with genotype as categorical factor. We applied the two-sample Kolmogorov–Smirnov (KS) test with Bonferroni correction to check for differences between the empirical distributions related to cortical and hippocampal EEG phase differences of control and Ts65Dn mice. Comparisons of mean levels of TBS-induced LTP in slices from a given genotype (control or Ts65Dn mice), subjected to 200 μM GABA, 1 μM diazepam, 10 μM diazepam, or aCSF only, were done by a one-way ANOVA. Comparisons of mean normalized levels of TBS-induced LTP between genotypes (control *versus* Ts65Dn mice), subjected to 200 μM GABA, 1 μM diazepam, 10 μM diazepam, or aCSF only, were performed by two-way ANOVA. Mean values of seizure severity score and latency were compared using two-way ANOVA, with genotype and treatment as categorical factors. Log-rank (Mantel-Cox) test was used to compare survival between groups. Mean values of spatiotemporal and ethological parameters were analyzed by two-way ANOVA, with genotype and treatment as categorical factors. Fisher’s least significant difference (LSD) post hoc analysis was performed when a genotype or treatment-dependence was detected by RM, one-, or two-way ANOVA. Values of *P* < 0.05 were considered statistically significant. P-values of < 0.05, 0.01, and 0.001 were represented in the figures by the symbols *, **, and ***, respectively. For sake of clarity and brevity, only *P* values were reported in the results section for ANOVA; F-statistics and their associated degrees of freedom can be found in “Supplementary data”.

## Supplementary Information

Supplementary Information.

## Data Availability

All other data are included in the supplemental information or will be made available upon requests.
