# Supplementary material for: Atypical electrophysiological and behavioral responses to diazepam in a leading mouse model of Down syndrome
Source: Sci Rep. 2021 May 4;11:9521. doi: 10.1038/s41598-021-89011-y (PMC8096846; doi:10.1038/s41598-021-89011-y)
Supplement: Supplementary file 1 — Supplementary Information. [file 41598_2021_89011_MOESM1_ESM.pdf]

Supplementary data for

Atypical electrophysiological and behavioral responses to diazepam in a leading mouse model of Down syndrome

Daniella B. Victorino, Daniel J. L. L. Pinheiro, Jonah J. Scott-McKean, Sarah Barker, Melissa R. Stasko, Jean Faber, Carla A. Scorza & Alberto C. S. Costa

Supplementary results

Diazepam induces significantly different changes on the neuronal activity spectral profile of control and Ts65Dn mice as recorded from a hippocampally-placed electrode. Similar to EEG analysis dataset from the cortical derivation, one-way MANOVA followed up with canonical discriminant analysis was employed to test whether diazapem-induced changes in EEG spectral power across multiple 2-Hz bands (0.5 to 50 Hz) in the hippocampal derivation could be used to differentiate control from Ts65Dn mice (Fig. S1b, c and d). Visual inspection of the grouped scatter plots of the first two canonical variables showed that *Can1* discriminated the control mice group from the Ts65Dn mice group at 3- and 10-mg/kg doses (Fig. S1c and d respectively). One-way ANOVA ($F_{(5, 33)} = 18.540$, $P < 0.0001$), followed by Fisher's LSD post hoc test ($P = 0.0031$ and $P < 0.0001$ at 3- and 10-mg/kg doses, respectively), confirmed these findings (Fig. S1e; Supplementary Tables S1 and S2). In contrast, *Can2* only differentiated control and Ts65Dn mice groups ($F_{(5, 33)} = 5.868$, $P = 0.0006$) at the 3-mg/kg dose ($P = 0.0001$; Fig. S1f; Supplementary Tables S1 and S2). A dendrogram representation of the Euclidian distances between group centroids (i.e., the group's mean) showed that the centroid of control mice group treated with 10 mg/kg diazepam was at the highest level of the hierarchical tree (Fig. S1g). In addition, the centroids for Ts65Dn mice groups treated with 1- and 10-mg/kg doses had the shortest distance among all centroids, followed by Ts65Dn mice group treated with 3-mg/kg dose (Fig. S1g), which means that Ts65Dn mice group clusters display a high between-cluster similarity. In contrast, the centroids for control mice groups treated with 1 and 3 mg/kg diazepam (as well as 10 mg/kg) are well separated, which reflects a low between-cluster similarity in such groups (Fig. S1g).

We then compared the normalized PSDs obtained from baseline and post-dosing recordings for each dose in each genotype (Fig. S1h-j, upper and middle portions) to determine the differences in diazepam effects between control and Ts65Dn mice for each experimental condition (Fig. S1h-j, lower portions). We

found that all doses of diazepam decreased EEG spectral power of slower brain rhythms in both control and Ts65Dn mice (Fig. S1h-j). A spectral power decrease in the 0.50-2.80 Hz was induced by 1 mg/kg diazepam in control mice (Fig. S1h, upper portion), whereas activity in the 0.60-2.20 Hz range was decreased in Ts65Dn mice (Fig. S1h, middle portion). At 3-mg/kg dose, diazepam decreased spectral power in the frequency range of 0.60 to 2.92 Hz and 0.73 to 2.92 Hz in control and Ts65Dn mice, respectively (Fig. S1i, upper and middle portions). Finally, diazepam at 10-mg/kg dose produced a significant power decrease in frequencies within the delta range in both control (0.50-3.29 Hz; Fig. S1j, upper portion) and Ts65Dn mice (0.50-2.80 Hz; Fig S1j, middle portion). We found a significant power decrease in frequencies within the delta range (2.31-3.66 Hz) in controls compared to Ts65Dn mice at 10-mg/kg dose (Fig. S1j, lower portion). Similarly, a significant difference in EEG spectral power within the theta frequency range of 6.46-7.20 Hz was also found between control and Ts65Dn mice at 10-mg/kg dose (Fig. S1j, lower portion).

Diazepam also affected EEG activity in frequency bins within alpha and beta ranges (Fig. S1h-j). A spectral power decrease in the 11.47 to 15.74 Hz, along with a spectral power increase in the 20.38-20.87 Hz, was induced by 1 mg/kg diazepam in control mice (Fig. S1h, upper portion). A significant difference in EEG spectral power within the alfa and beta frequency ranges (11.59 to 15.74 Hz and 20.50 to 21.97 Hz) was found between control and Ts65Dn mice at 1-mg/kg dose (Fig. S1h, lower portion). At 10-mg/kg dose, diazepam decreased spectral power in the frequency range of 9.52 to 12.57 Hz in Ts65Dn mice (Fig. S1j, middle portion).

Finally, fast activities in control and Ts65Dn mice were also differently affected by diazepam. At 1-mg/kg dose, diazepam increased spectral power in the frequency range of 44.92-47.48 Hz in Ts65Dn mice (Fig. S1h, middle portion). We also found that 3 mg/kg diazepam decreased spectral power in the frequency range of 28.68-35.27 Hz in Ts65Dn mice (Fig. S1i, middle portion). Diazepam at 10-mg/kg dose produced a significant spectral power decrease within the 33.32-36.01 Hz frequency range in Ts65Dn mice (Fig. S1j, middle portion). A significant difference in EEG spectral power in the 30.27-31.25 Hz was found between control and Ts65Dn mice at 10-mg/kg dose (Fig. S1j, lower portion).

Diazepam-induced changes in the pattern of spectral power distribution between control and Ts65Dn mice showed subtle, but significant differences. We found that the effects of diazepam on the pattern of spectral power distribution were limited to delta and theta frequency bands (Fig. S3a-d). At 1-

mg/kg, diazepam ($F_{(1, 11)} = 20.739$, $P = 0.0008$; Supplementary Table S3) significantly increased the skewness of the data distribution within the delta band in both control and Ts65Dn mice. Post hoc tests identified significant differences between the mean skewness values for both control and Ts65Dn mice compared with their respective baseline conditions ($P = 0.0026$ and $P = 0.0288$; respectively; Fig. S3e; Supplementary Table S5). The observed diazepam-induced increase ($F_{(1, 11)} = 11.952$, $P = 0.0054$; Supplementary Table S3) in the mean frequency within the delta band (i.e., the mean of the distribution increases as skewness becomes more positive) corroborates with such a finding. The observed increase in the mean frequency within the delta band was significant for both control and Ts65Dn mice compared with their respective baseline condition ($P = 0.0482$ and $P = 0.0211$; respectively; Fig. S3f; Supplementary Table S5). Our findings suggest that the pattern of neuronal activity induced by 1 mg/kg diazepam within the delta band followed a positively skewed distribution, wherein the mass of the data was concentrated on the left side of the mean point. Such observation could be interpreted as a diazepam-induced shift of the PSD curve toward lower frequency values, which might suggest a slowing effect of diazepam on EEG frequencies[1, 2, 3, 4]. Consistently, we also observed that 1 mg/kg diazepam increased the mean values of kurtosis within the delta band ($F_{(1, 11)} = 10.852$, $P = 0.0072$; Supplementary Table S3). Fisher's post hoc tests confirmed only treatment-dependent differences between diazepam-treated control mice and their baseline condition ($P = 0.0069$; Fig. S3g; Supplementary Table S5). This observation indicates an increase in the tail heaviness of the data distribution, which parallels the findings of an increased skewness.

Finally, diazepam at 1-mg/kg ($F_{(1, 11)} = 11.528$, $P = 0.0060$; Supplementary Table S3) significantly increased the skewness of the data distribution within the theta band in control mice, but this effect of diazepam was not detected in Ts65Dn mice. A significant interaction between treatment and genotype was also found ($F_{(1, 11)} = 12.663$, $P = 0.0045$). Diazepam-treated control mice showed a significant increase in the skewness of the data distribution within the theta band compared to both its baseline condition ($P = 0.0006$) and diazepam-treated Ts65Dn mice ($P = 0.0056$; Fig. S3h; Supplementary Table S5). Such diazepam-induced left side shift of data distribution was accompanied by a decrease in the power of the mean frequency ($F_{(1, 11)} = 5.942$, $P = 0.0330$; Supplementary Table S3). Fisher's post hoc tests confirmed that the observed decrease in the mean frequency power of the theta band was significant for diazepam-treated control mice when compared with its baseline condition ($P = 0.0230$; Fig. S3i; Supplementary Table S5).

Finally, we found that diazepam at 1-mg/kg ($F_{(1, 11)} = 5.540$, $P = 0.0382$; Supplementary Table S3) significantly increased the mean values of kurtosis within the theta band. A significant interaction was also detected between treatment and genotype ($F_{(1, 11)} = 7.662$, $P = 0.0183$). Post hoc tests confirmed that diazepam-treated control mice showed a significant increase in the mean kurtosis values compared to both its baseline condition ($P = 0.0051$) and diazepam-treated Ts65Dn mice ($P = 0.0067$; Fig. S3j; Supplementary Table S5). Together, these findings suggest that a slowing effect of 1 mg/kg diazepam on EEG frequencies within delta and theta frequency ranges. While this effect was similar between control and Ts65Dn mice in the delta band, it occurred in a genotype-dependent manner within the theta band. In addition, the effects on the kurtosis within both delta and theta bands were only found for the control mice. Therefore, we speculate that the slowing effect of diazepam on EEG frequencies is diminished in Ts65Dn mice.

Neither GABA nor diazepam affected input/output (I/O) curves and paired-pulse facilitation (PPF) in both Ts65Dn and control mice. To investigate the effects of GABA (200 μM) and diazepam (1 and 10 μM) on basic synaptic function of both control and Ts65Dn mice, we assessed I/O function for hippocampal slices treated either with aCSF, 200 μM of GABA, 1 μM or 10 μM of diazepam. For control mice (Fig. S4a), analysis of treatment effect on I/O function by RM ANOVA showed no significant differences ($F_{(3, 44)} = 0.113$, $P = 0.9523$). In addition, no significant interaction was found between treatment and stimulus intensity ($F_{(27, 396)} = 0.238$, $P = 0.9999$). On the other hand, RM ANOVA revealed a significant stimulus intensity effect on I/O function ($F_{(9, 396)} = 147.788$, $P < 0.001$; Supplementary Table S10). For Ts65Dn mice (Fig. S4b), analysis of the I/O function by RM ANOVA showed neither significant treatment effect ($F_{(3, 44)} = 0.416$, $P = 0.7425$) nor interaction between treatment and stimulus intensity ($F_{(27, 396)} = 0.258$, $P = 0.9999$). In contrast, there was also a significant stimulus intensity effect on I/O function ($F_{(9, 396)} = 228.287$, $P < 0.001$; Supplementary Table S10).

We also assessed the effects of GABA (200 μM) and diazepam (1 and 10 μM) on PPF, which is a type of short-lived plasticity sensitive to presynaptic changes. To evaluate the integrity of presynaptic mechanisms, we induced PPF in control- and Ts65Dn-derived hippocampal slices by delivering two stimuli of identical strength at different interpulse intervals. The slope ratio between the second and first stimuli was

then used to calculate the PPF. For control mice (Fig. S4c), RM ANOVA showed no significant treatment effect ($F_{(3, 44)} = 0.838$, $P = 0.4802$) or interaction between treatment and interpulse interval ($F_{(21, 308)} = 1.265$, $P = 0.1967$). In contrast, a significant interpulse interval effect was found ($F_{(7, 308)} = 365.632$, $P < 0.001$; Supplementary Table S10). As with control mice, analysis of the data for Ts65Dn mice (Fig. S4d) showed no significant treatment effect ($F_{(3, 44)} = 0.360$, $P = 0.7824$) or interaction between treatment and interpulse interval ($F_{(21, 308)} = 0.950$, $P = 0.5261$). We also found a significant interpulse interval effect ($F_{(7, 308)} = 336.410$, $P < 0.001$; Supplementary Table S10).

Ts65Dn and control mice show differential sensitivity to diazepam modulation of anxiety-like behavioral responses. Two-way ANOVA indicated that neither genotype ($F_{(1, 56)} = 0.077$, $P = 0.7818$) nor treatment ($F_{(1, 56)} = 3.831$, $P = 0.0553$; Fig. S5a; Supplementary Table S13) had any significant effect on the mean values of closed arm entries. Also, no significant interaction was found between genotype and treatment ($F_{(1, 56)} = 0.127$, $P = 0.7227$). Similarly, analysis of percentage of time spent in closed arms by mice revealed no significant genotype ($F_{(1, 56)} = 0.980$, $P = 0.3264$) and treatment-dependent differences ($F_{(1, 56)} = 1.041$, $P = 0.3119$; Fig. S5b; Supplementary Table S13). No significant interaction between genotype and treatment ($F_{(1, 56)} = 0.004$, $P = 0.9525$) was found. Finally, two-way ANOVA showed that the percentage of time spent in the center of the platform was not significantly affected by either genotype ($F_{(1, 56)} = 0.111$, $P = 0.7404$) or treatment ($F_{(1, 56)} = 3.920$, $P = 0.0526$), and a significant interaction between genotype and treatment ($F_{(1, 56)} = 0.501$, $P = 0.4822$) was not detected (Fig. S5c; Supplementary Table S13).

We also evaluated the frequency of closed arm returns, which is characterized by the animal exiting a closed arm with both forepaws and immediately returning into the closed arm. Two-way ANOVA showed that genotype ($F_{(1, 56)} = 5.833$, $P = 0.0190$), but not treatment ($F_{(1, 56)} = 1.529$, $P = 0.2214$; Supplementary Table S13) had significant effects on the mean values of this parameter. Also, no significant interaction was found between genotype and treatment ($F_{(1, 56)} = 0.860$, $P = 0.3577$). Fisher's LSD post hoc test confirmed that vehicle-treated control mice were more reluctant to leave the closed arm than vehicle-treated Ts65Dn mice ($P = 0.0216$; Fig. S5d; Supplementary Table S14).

Finally, we found that genotype ($F_{(1, 56)} = 6.359$, $P = 0.0145$), but not treatment ($F_{(1, 56)} = 2.224$, $P = 0.1415$; Supplementary Table S13) had significant effects on the mean values of time of grooming. No

significant interaction was found between genotype and treatment ($F_{(1, 56)}$ = 0.025, P = 0.8748). Post hoc tests failed to detect any significant differences in the time of grooming between control and Ts65Dn mice treated with vehicle (P = 0.1003) as well as between diazepam-treated control and Ts65Dn mice (P = 0.0633; Fig. S4e; Supplementary Table S14).

References

1. Cramer NP, Xu X, T FH, Galdzicki Z. Altered intrinsic and network properties of neocortical neurons in the Ts65Dn mouse model of Down syndrome. *Physiol Rep* **3**, (2015).

2. Kuki T, Fujihara K, Miwa H, Tamamaki N, Yanagawa Y, Mushiake H. Contribution of parvalbumin and somatostatin-expressing GABAergic neurons to slow oscillations and the balance in beta-gamma oscillations across cortical layers. *Front Neural Circuits* **9**, 6 (2015).

3. Luczak A, Bartho P, Marguet SL, Buzsaki G, Harris KD. Sequential structure of neocortical spontaneous activity in vivo. *Proc Natl Acad Sci U S A* **104**, 347-352 (2007).

4. Rijn CM, Jongsma M. Chronic effects of diazepam on the spectral content of the rat EEG. (ed^(eds) (1995).

Supplementary Figures

Figure S1

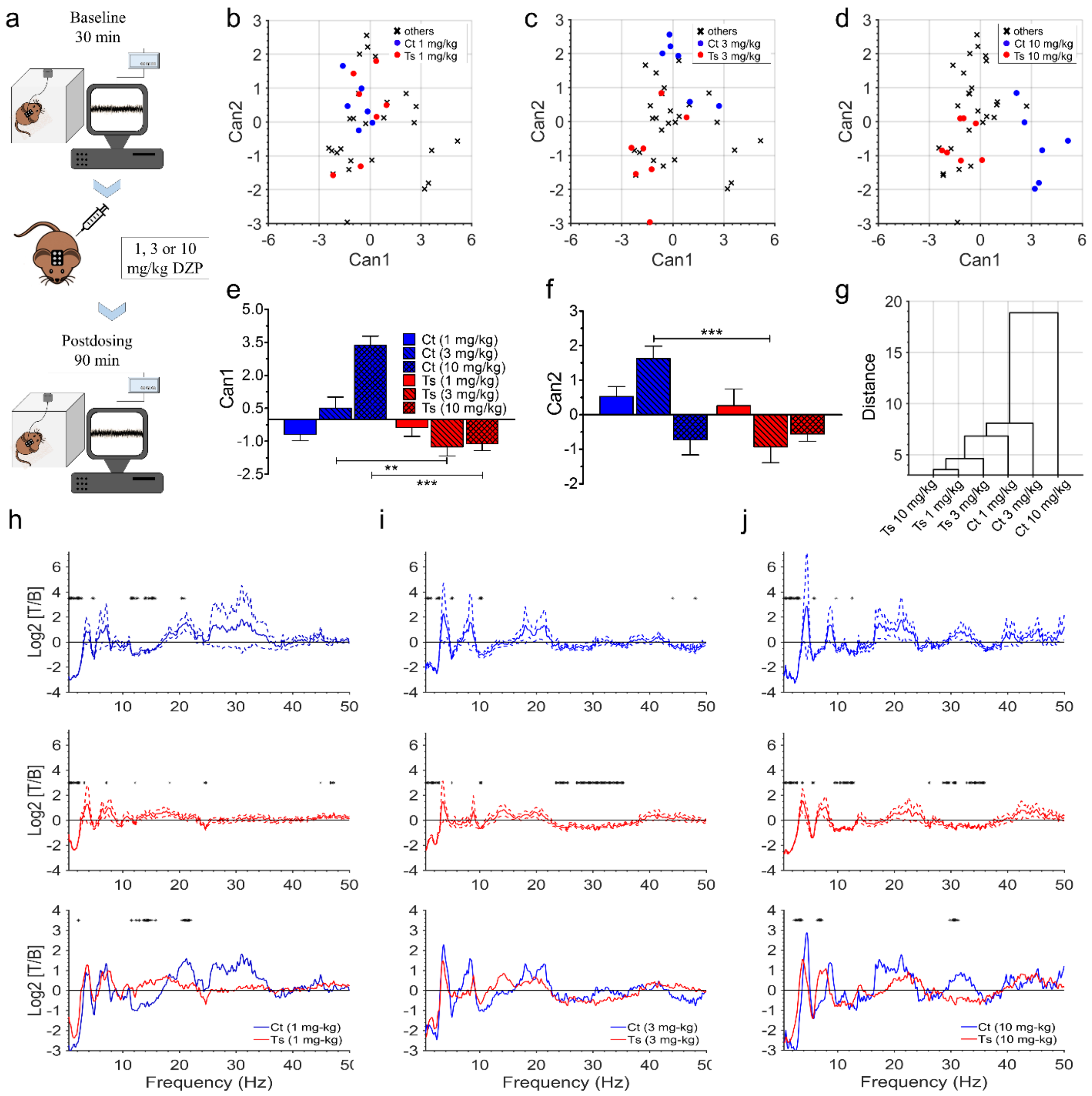

Fig. S1: Diazepam induces significantly different changes on the neuronal activity spectral profile of control and Ts65Dn mice as recorded from a hippocampally-placed electrode. a Freely moving mice implanted with EEG electrodes were connected through a cable to the recording unit. The experiment consisted of a 30-min baseline recording, followed by a 90-min post-dosing recording, in which mice were acutely treated with 1, 3, or 10 mg/kg diazepam. **b-d** Grouped scatter plots of the first two canonical variables for all mice analyzed are shown in **b**, **c**, and **d** for 1-, 3-, and 10-mg/kg doses of diazepam, respectively. Colored points represent mice and are labelled by cluster group. **e** *Can1* discriminated the control mice group from the Ts65Dn mice group at 3- and 10-mg/kg doses. **f** *Can2* only differentiated control and Ts65Dn mice groups at the 3-mg/kg dose. **g** Dendrogram revealed that the centroid of control mice group treated with 10 mg/kg diazepam was at the highest level of the hierarchical tree, whereas the centroids of Ts65Dn mice groups treated with 1- and 10-mg/kg doses had the shortest distance among all centroids (the height of each pair of branches represents the Euclidian distances between group centroids). **h-j** PSD obtained from baseline and post-dosing recordings were compared for each dose in each genotype (black asterisks in upper and middle panels represent frequency bins in which significant differences in EEG spectral power were observed between baseline and treatment conditions; relative changes are expressed as the $\log_2$ of the treatment/baseline ratio). Normalized PSD of control and Ts65Dn mice for each experimental condition was also compared (lower panels). Number of mice: Ct ($N = 6$) and Ts ($N = 7$) for all experimental conditions. In **e** and **f**, data represent mean ± SEM and statistical significance is expressed as ** and *** for $P < 0.01$ and $P < 0.001$, respectively. In **h-j**, solid lines represent means and dashed lines represent SEM and black asterisks indicate statistical significance ($P < 0.05$).

Figure S2

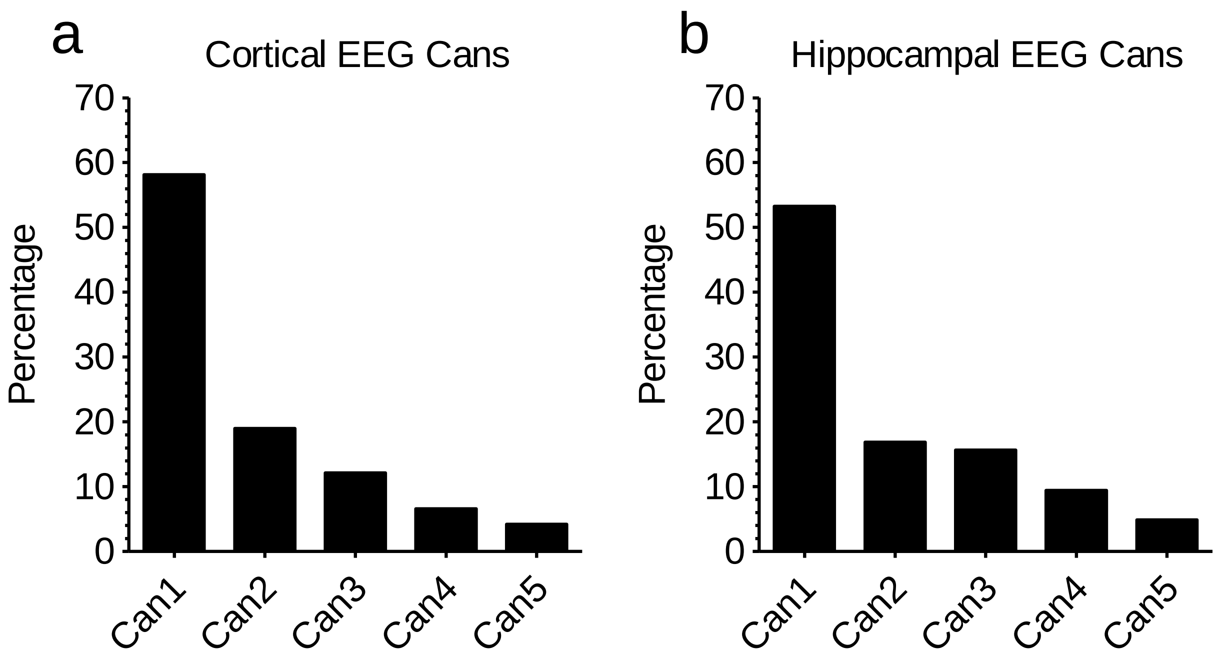

Fig. S2: Relative Sizes of the Canonical Variables. a and **b** are bargraph representations of the relative sizes of the different *Can* components of the acartical and hippocampal EEGs. These relative sizes summarize between-class variation in much the same way that principal components summarize total variation. As should be expected for this type of analysis, the relative size of *Can1* (58.14%, and 53.24%, for cortex and hippocampus respectively) is much larger than *Can2* (18.96%, and 16.85%, for cortex and hippocampus respectively) or of any calculated higher order canonical variables.

Figure S3

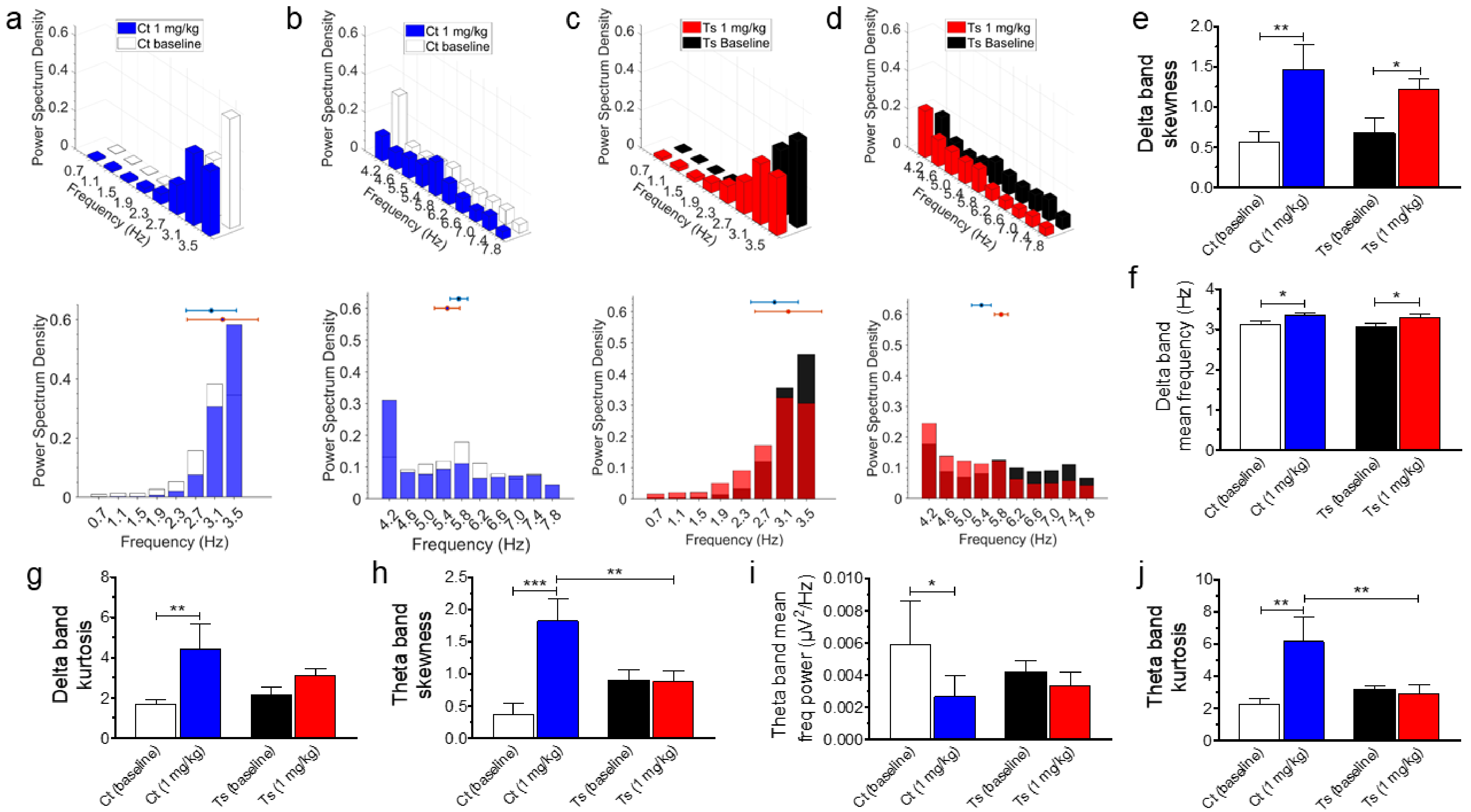

a
b
c
d
e
f
g
h
i
j
Ct 1 mg/kg
Ct baseline
Ts 1 mg/kg
Ts Baseline
Power Spectrum Density
Frequency (Hz)
Delta band skewness
Delta band mean frequency (Hz)
Delta band kurtosis
Theta band skewness
Theta band mean freq power (µV²/Hz)
Theta band kurtosis
Ct (baseline)
Ct (1 mg/kg)
Ts (baseline)
Ts (1 mg/kg)

Fig. S3: Diazepam induces a shift of the PSD curve toward lower frequency values in both control and Ts65Dn mice, with a relatively smaller effect seen in Ts65Dn vs control euploid mice. To verify whether diazepam induces a slowing effect on neuronal oscillations, frequency histograms associated with each rhythm were calculated and compared with respect to their third and fourth statistical moments (i.e., skewness and kurtosis). Fig. S3 illustrates how skewness and kurtosis of frequency distribution within delta and theta bands vary between baseline and post-dosing recordings. **a-d** The effects of 1 mg/kg diazepam on the pattern of spectral power distribution were limited to delta (**a** for control and **b** for Ts65Dn mice) and theta (**c** for control and **d** for Ts65Dn mice) frequency bands. The height of each bar in the histograms represents PSD. Each 0.4 Hz-size class of the histogram represents EEG frequencies (Hz). Solid circles at the top of each 2D histogram represent mean frequency and solid lines represent SEM. **e** Diazepam at 1-mg/kg dose significantly increased mean skewness values within the delta band in both control and Ts65Dn mice compared with their respective baseline conditions. **f** A significant increase in the mean frequency within the delta band was also observed for both diazepam-treated control and Ts65Dn mice compared with their respective baseline condition. **g** Diazepam at 1-mg/kg dose also significantly increased mean kurtosis values within the delta band, **h** as well as mean skewness values within the theta band in control mice compared with its respective baseline condition. Diazepam-treated control mice showed significantly increased mean skewness values within the theta band compared to diazepam-treated Ts65Dn mice. **i** A significant decrease in the mean frequency power of the theta band was observed for diazepam-treated control mice compared with its baseline condition. **j** Diazepam at 1-mg/kg dose also significantly increased the mean kurtosis values within the theta band in control mice compared with its respective baseline condition, as well as with diazepam-treated Ts65Dn mice. Number of mice: Ct baseline (N = 6), Ct 1 mg/kg diazepam (N = 6), Ts baseline (N = 7), and Ts 1 mg/kg diazepam (N = 7). Data represent mean ± SEM. Statistical significance is expressed as *, ** and *** for $P < 0.05$, $P < 0.01$, and $P < 0.001$, respectively.

Figure S4

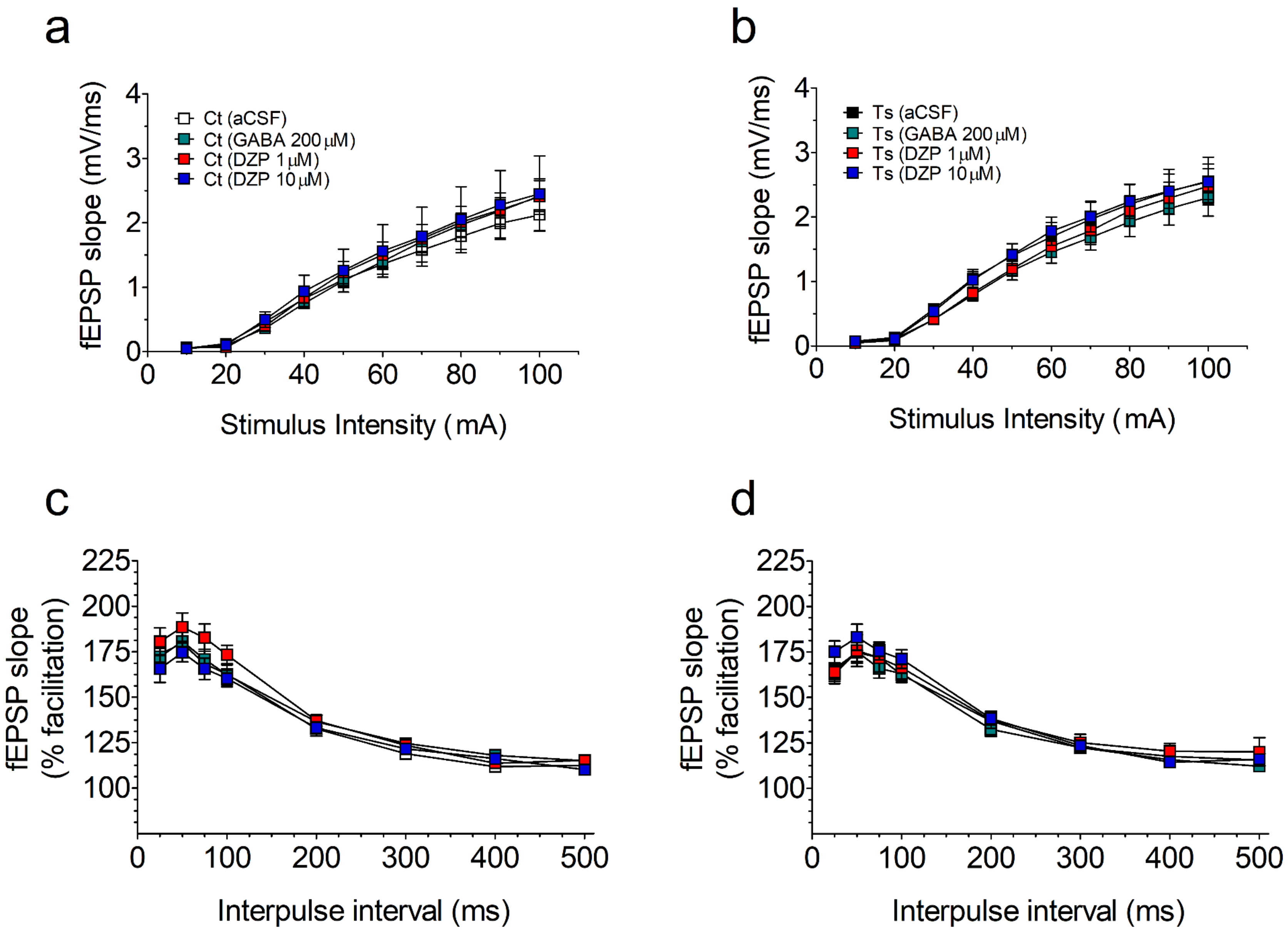
a
b
c
d
fEPSP slope (mV/ms)
Stimulus Intensity (mA)
Ct (aCSF)
Ct (GABA 200 μM)
Ct (DZP 1 μM)
Ct (DZP 10 μM)
Ts (aCSF)
Ts (GABA 200 μM)
Ts (DZP 1 μM)
Ts (DZP 10 μM)
fEPSP slope (% facilitation)
Interpulse interval (ms)

Fig. S4: Neither GABA nor diazepam had an effect on either basal synaptic transmission or presynaptic mechanism integrity in both control and Ts65Dn mice. I/O function at the CA3–CA1 hippocampal synapse was assessed for control- (**a**) and Ts65Dn- (**b**) derived hippocampal slices treated with either aCSF, GABA (200 μM) or diazepam (1 and 10 μM). Input intensities were set at 10, 20, 30, 40, 50, 60, 70, 80, 90, and 100 μA. I/O curves were not affected by either GABA (200 μM) or diazepam (1 and 10 μM) in both control (**a**) and Ts65Dn mice (**b**). PPF was also assessed for control- (**c**) and Ts65Dn- (**d**) derived hippocampal slices treated with either aCSF, GABA (200 μM) or diazepam (1 and 10 μM). PPF was evaluated at eight different interpulse intervals: 25, 50, 75, 100, 200, 300, 400, and 500 msec. As with I/O function, PPF was not affected by either GABA (200 μM) or diazepam (1 and 10 μM) in both control (**c**) and Ts65Dn mice (**d**). Number of slices/mice: Ct aCSF (N = 12), Ct 200 μM GABA (N = 12), Ct 1 μM diazepam (N = 12), Ct 10 μM diazepam (N = 12), Ts aCSF (N = 12), Ts 200 μM GABA (N = 12), Ts 1 μM diazepam (N = 12), and Ts 10 μM diazepam (N = 12). Data represent mean ± SEM.

Figure S5

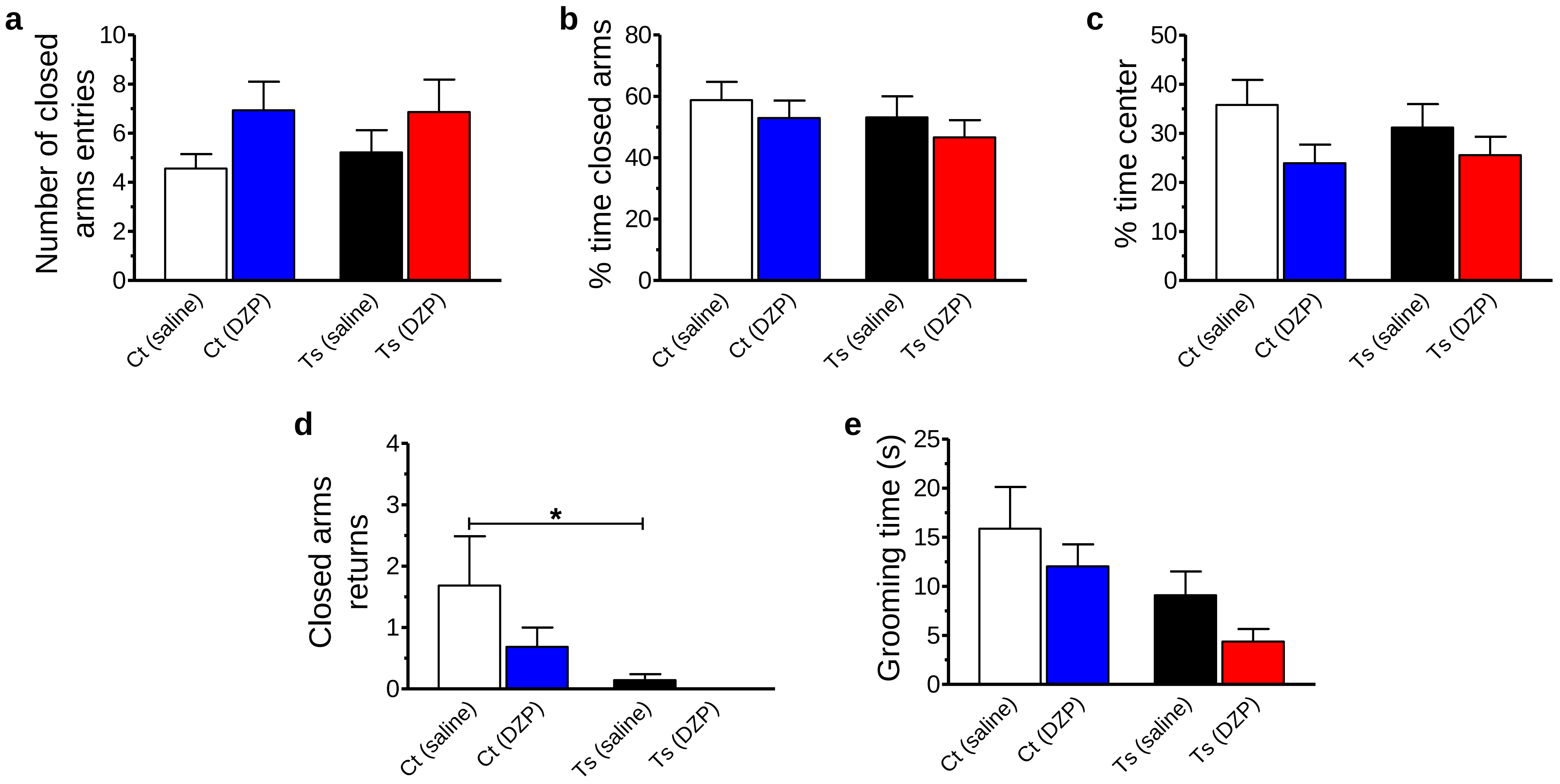

Fig. S5: Ts65Dn and control mice exhibit a similar pattern of exploratory behavior, but vehicle-treated Ts65Dn mice had less closed arms returns than vehicle-treated control mice. a Diazepam at 1-mg/kg dose did not affect the number of closed arms entries, **b** as well as the percentage of time spent in closed

arms in both control and Ts65Dn mice. **c** The percentage of time spent in the center of the platform by diazepam-treated control and Ts65Dn mice was also not affected. **d** Vehicle treated-Ts65Dn mice showed significantly less closed arm returns compared with vehicle-treated control mice. **e** Time of grooming in either control or Ts65Dn mice was not affected by 1 mg/kg diazepam. Number of mice: Ct baseline (N = 16), Ct 1 mg/kg diazepam (N = 16), Ts baseline (N = 14), and Ts 1 mg/kg diazepam (N = 14). Data represent mean ± SEM. Statistical significance is expressed as * for $P < 0.05$.

Supplementary Tables

Table S1. Canonical discriminant analysis (cortical and hippocampal derivations)

Canonical discriminant analysis (*Can*)					
One-way Analysis of Variance					
Canonical variables	Brain area	Effect	Degr. of (Freedom)	F	P value
Can1	Cortex	Intercept	1	0.376	0.5440
		Group	5	25.590	0.0000
		Error	33		
	Hippocampus	Intercept	1	0.224	0.6393
		Group	5	18.540	0.0000
		Error	33		
Can2	Cortex	Intercept	1	0.067	0.7975
		Group	5	8.346	0.0000
		Error	33		
	Hippocampus	Intercept	1	0.045	0.8338
		Group	5	5.868	0.0006
		Error	33		

Table S2. Fisher's least significant difference (LSD) post hoc analysis - Canonical variables (cortical and hippocampal derivations)

Canonical discriminant analysis (*Can*)			
Fisher's LSD post hoc analysis			
Group/Treatment	Brain area	Canonical variables	P value
Control (1 mg/kg DZP) *vs* Ts65Dn (1 mg/kg DZP)	Cortex	*Can*1	0.0009
		*Can*2	0.0003
	Hippocampus	*Can*1	0.5836
		*Can*2	0.6363
Control (3 mg/kg DZP) *vs* Ts65Dn (3 mg/kg DZP)	Cortex	*Can*1	0.0055
		*Can*2	0.0019
	Hippocampus	*Can*1	0.0031
		*Can*2	0.0001
Control (10 mg/kg DZP) *vs* Ts65Dn (10 mg/kg DZP)	Cortex	*Can*1	0.0000
		*Can*2	0.1147
	Hippocampus	*Can*1	0.0000
		*Can*2	0.7611

Table S3. Analysis of spectral parameters - cortical derivation

Spectral parameters - cortical derivation									
Repeated Measures Analysis of Variance									
Waveband	Spectral parameter	Effect	Degr. of (Freedom)	Dose (mg/kg)					
				1		3		10	
				F	P value	F	P value	F	P value
Delta	Band power	Intercept	1	187.256	0.0000	32.222	0.0001	46.640	0.0000
		Genotype	1	0.127	0.7281	2.023	0.1827	0.094	0.7650
		Error	11						
		Treatment	1	0.389	0.5458	1.190	0.2986	0.259	0.6208
		Treatment*Genotype	1	0.040	0.8457	0.147	0.7089	1.383	0.2645
		Error	11					0.002	
	Mean frequency	Intercept	1	6420.763	0.0000	18087.465	0.0000	7802.176	0.0000
		Genotype	1	0.424	0.5282	1.164	0.3038	0.656	0.4351
		Error	11						
		Treatment	1	11.952	0.0054	19.736	0.0010	24.195	0.0005
		Treatment*Genotype	1	0.039	0.8476	0.177	0.6819	0.483	0.5013
		Error	11						
	Power mean frequency	Intercept	1	43.819	0.0000	13.585	0.0036	19.213	0.0011
		Genotype	1	0.463	0.5103	1.437	0.2559	0.221	0.6477
		Error	11						
		Treatment	1	0.550	0.4737	2.805	0.1221	4.804	0.0508
		Treatment*Genotype	1	0.021	0.8887	0.211	0.6552	1.059	0.3256
		Error	11						
	Skewness	Intercept	1	78.127	0.0000	138.783	0.0000	243.118	0.0000
		Genotype	1	0.083	0.7788	2.475	0.1440	0.018	0.8943
		Error	11						
		Treatment	1	20.739	0.0008	11.300	0.0063	38.340	0.0001
		Treatment*Genotype	1	1.294	0.2795	1.917	0.1937	0.110	0.7464
		Error	11						
	Kurtosis	Intercept	1	59.763	0.0000	56.640	0.0000	106.850	0.0000
		Genotype	1	0.292	0.6000	2.376	0.1515	0.019	0.8926
		Error	11						
		Treatment	1	10.852	0.0072	6.347	0.0285	19.902	0.0010

		Treatment*Genotype	1	2.454	0.1455	2.460	0.1451	0.175	0.6837
		Error	11						
Theta	Band power	Intercept	1	80.156	0.0000	102.589	0.0000	139.958	0.0000
		Genotype	1	0.335	0.5745	0.593	0.4576	0.009	0.9264
		Error	11						
		Treatment	1	1.914	0.1940	0.069	0.7981	2.452	0.1457
		Treatment*Genotype	1	1.750	0.2127	0.167	0.6905	0.074	0.7910
		Error	11						
	Mean frequency	Intercept	1	5759.644	0.0000	4970.124	0.0000	2714.877	0.0000
		Genotype	1	0.262	0.6190	0.506	0.4918	0.092	0.7673
		Error	11						
		Treatment	1	1.917	0.1936	0.000	0.9926	2.701	0.1285
		Treatment*Genotype	1	8.566	0.0138	0.061	0.8095	1.086	0.3198
		Error	11						
	Power mean frequency	Intercept	1	17.706	0.0015	10.684	0.0075	14.624	0.0028
		Genotype	1	0.079	0.7842	0.862	0.3732	3.710	0.0803
		Error	11						
		Treatment	1	5.942	0.0330	3.562	0.0858	0.946	0.3516
		Treatment*Genotype	1	2.063	0.1787	1.768	0.2106	0.979	0.3437
		Error	11						
	Skewness	Intercept	1	77.053	0.0000	62.303	0.0000	105.180	0.0000
		Genotype	1	0.795	0.3918	1.500	0.2462	1.112	0.3142
		Error	11						
		Treatment	1	11.528	0.0060	3.781	0.0779	4.930	0.0483
		Treatment*Genotype	1	12.663	0.0045	0.092	0.7673	0.001	0.9791
		Error	11						
	Kurtosis	Intercept	1	85.926	0.0000	60.475	0.0000	129.655	0.0000
		Genotype	1	2.200	0.1661	0.525	0.4840	2.358	0.1529
		Error	11						
		Treatment	1	5.540	0.0382	5.551	0.0381	4.212	0.0647
		Treatment*Genotype	1	7.662	0.0183	0.101	0.7563	0.125	0.7307

		Error	11						
Alfa	Band power	Intercept	1	84.107	0.0000	221.523	0.0000	53.590	0.0000
		Genotype	1	0.163	0.6943	0.230	0.6411	1.759	0.2116
		Error	11						
		Treatment	1	0.146	0.7098	1.772	0.2100	2.540	0.1393
		Treatment*Genotype	1	0.048	0.8314	0.047	0.8330	3.411	0.0918
		Error	11						
	Mean frequency	Intercept	1	49895.606	0.0000	19597.536	0.0000	16445.045	0.0000
		Genotype	1	0.910	0.3605	0.607	0.4522	0.555	0.4719
		Error	11						
		Treatment	1	0.542	0.4768	1.096	0.3177	0.086	0.7742
		Treatment*Genotype	1	1.056	0.3263	2.927	0.1151	0.004	0.9509
		Error	11						
	Power mean frequency	Intercept	1	47.714	0.0000	52.874	0.0000	19.864	0.0010
		Genotype	1	0.095	0.7633	0.578	0.4632	1.784	0.2087
		Error	11						
		Treatment	1	0.367	0.5572	0.391	0.5443	3.069	0.1076
		Treatment*Genotype	1	0.013	0.9107	0.136	0.7190	3.542	0.0865
		Error	11						
	Skewness	Intercept	1	33.236	0.0001	21.551	0.0007	29.348	0.0002
		Genotype	1	3.177	0.1023	4.053	0.0692	0.033	0.8593
		Error	11						
		Treatment	1	0.023	0.8820	0.047	0.8320	0.047	0.8319
		Treatment*Genotype	1	0.880	0.3683	0.217	0.6501	0.710	0.4174
		Error	11						
	Kurtosis	Intercept	1	98.199	0.0000	105.255	0.0000	108.094	0.0000
		Genotype	1	2.718	0.1275	0.815	0.3859	0.007	0.9349
		Error	11						
		Treatment	1	0.041	0.8424	1.989	0.1861	1.017	0.3348
		Treatment*Genotype	1	0.535	0.4797	1.569	0.2363	5.603	0.0373
		Error	11						

Beta 1	Band power	Intercept	1	70.721	0.0000	65.662	0.0000	122.956	0.0000
		Genotype	1	5.350	0.0411	0.001	0.9816	2.221	0.1643
		Error	11						
		Treatment	1	5.496	0.0389	0.055	0.8195	0.503	0.4927
		Treatment*Genotype	1	0.927	0.3563	0.057	0.8150	3.014	0.1105
		Error	11						
	Mean frequency	Intercept	1	107048.220	0.0000	193908.874	0.0000	99613.372	0.0000
		Genotype	1	0.138	0.7178	0.694	0.4226	1.522	0.2430
		Error	11						
		Treatment	1	0.001	0.9821	6.078	0.0314	0.642	0.4399
		Treatment*Genotype	1	1.541	0.2402	0.006	0.9377	2.550	0.1386
		Error	11						
	Power mean frequency	Intercept	1	45.511	0.0000	37.584	0.0001	94.907	0.0000
		Genotype	1	4.027	0.0700	0.050	0.8271	1.739	0.2140
		Error	11						
		Treatment	1	4.835	0.0502	0.952	0.3501	0.446	0.5179
		Treatment*Genotype	1	0.587	0.4598	0.003	0.9586	3.523	0.0873
		Error	11						
	Skewness	Intercept	1	17.797	0.0014	5.607	0.0373	12.601	0.0046
		Genotype	1	0.206	0.6588	0.033	0.8597	0.180	0.6797
		Error	11						
		Treatment	1	1.059	0.3255	2.260	0.1609	0.321	0.5827
		Treatment*Genotype	1	2.517	0.1409	0.185	0.6750	5.841	0.0342
		Error	11						
	Kurtosis	Intercept	1	59.515	0.0000	56.195	0.0000	102.960	0.0000
		Genotype	1	0.898	0.3637	0.135	0.7201	1.674	0.2222
		Error	11						
		Treatment	1	2.034	0.1816	2.452	0.1457	0.010	0.9216
		Treatment*Genotype	1	0.913	0.3599	1.260	0.2856	2.835	0.1204
		Error	11						
Beta 2	Band power	Intercept	1	389.480	0.0000	239.980	0.0000	206.068	0.0000

		Genotype	1	0.766	0.4002	0.008	0.9315	0.013	0.9111
		Error	11						
		Treatment	1	9.472	0.0105	0.228	0.6427	0.027	0.8736
		Treatment*Genotype	1	5.307	0.0418	2.718	0.1275	0.655	0.4356
		Error	11						
	Mean frequency	Intercept	1	15192.565	0.0000	11014.450	0.0000	6894.009	0.0000
		Genotype	1	0.203	0.6612	2.274	0.1597	0.003	0.9581
		Error	11						
		Treatment	1	2.263	0.1606	3.708	0.0804	0.037	0.8501
		Treatment*Genotype	1	0.001	0.9773	0.226	0.6437	0.106	0.7513
		Error	11						
	Power mean frequency	Intercept	1	175.772	0.0000	44.483	0.0000	74.029	0.0000
		Genotype	1	6.323	0.0288	0.697	0.4214	0.002	0.9689
		Error	11						
		Treatment	1	12.268	0.0049	4.977	0.0474	1.909	0.1944
		Treatment*Genotype	1	6.537	0.0267	4.913	0.0487	0.113	0.7433
		Error	11						
	Skewness	Intercept	1	50.901	0.0000	41.174	0.0000	40.302	0.0001
		Genotype	1	7.732	0.0179	0.463	0.5101	1.665	0.2233
		Error	11						
		Treatment	1	0.010	0.9235	8.248	0.0152	0.785	0.3947
		Treatment*Genotype	1	0.001	0.9764	0.001	0.9790	0.151	0.7053
		Error	11						
	Kurtosis	Intercept	1	44.799	0.0000	55.136	0.0000	76.668	0.0000
		Genotype	1	5.625	0.0370	0.383	0.5484	2.940	0.1144
		Error	11						
		Treatment	1	0.538	0.4787	3.091	0.1065	0.258	0.6212
		Treatment*Genotype	1	0.012	0.9145	0.451	0.5159	0.052	0.8239
		Error	11						
Gamma	Band power	Intercept	1	234.446	0.0000	1351.595	0.0000	220.007	0.0000
		Genotype	1	0.143	0.7128	11.012	0.0069	0.265	0.6171

		Error	11						
		Treatment	1	3.307	0.0963	5.397	0.0403	6.832	0.0241
		Treatment*Genotype	1	2.048	0.1802	0.001	0.9796	0.472	0.5061
		Error	11						
	Mean frequency	Intercept	1	34450.975	0.0000	29628.629	0.0000	37978.722	0.0000
		Genotype	1	0.350	0.5660	0.022	0.8835	0.332	0.5763
		Error	11						
		Treatment	1	1.124	0.3118	0.002	0.9641	1.834	0.2028
		Treatment*Genotype	1	0.033	0.8601	1.362	0.2679	4.340	0.0614
		Error	11						
	Power mean frequency	Intercept	1	106.462	0.0000	86.875	0.0000	154.719	0.0000
		Genotype	1	0.152	0.7042	0.071	0.7952	0.255	0.6233
		Error	11						
		Treatment	1	4.677	0.0535	1.395	0.2625	6.015	0.0321
		Treatment*Genotype	1	1.007	0.3372	0.109	0.7470	0.979	0.3436
		Error	11						
	Skewness	Intercept	1	22.131	0.0006	35.399	0.0001	95.668	0.0000
		Genotype	1	3.587	0.0848	0.548	0.4748	13.787	0.0034
		Error	11						
		Treatment	1	0.148	0.7075	0.007	0.9332	3.582	0.0850
		Treatment*Genotype	1	0.148	0.7081	0.077	0.7865	4.172	0.0658
		Error	11						
	Kurtosis	Intercept	1	56.846	0.0000	75.933	0.0000	100.003	0.0000
		Genotype	1	3.419	0.0915	1.187	0.2992	9.381	0.0108
		Error	11						
		Treatment	1	0.468	0.5079	0.040	0.8446	1.547	0.2395
		Treatment*Genotype	1	1.959	0.1892	1.626	0.2285	2.335	0.1547
		Error	11						
High gamma	Band power	Intercept	1	206.791	0.0000	258.661	0.0000	251.430	0.0000
		Genotype	1	0.438	0.5218	1.084	0.3201	0.027	0.8732
		Error	11						

	Treatment	1	2.681	0.1298	2.210	0.1652	0.819	0.3847
	Treatment*Genotype	1	1.058	0.3258	3.772	0.0781	0.024	0.8801
	Error	11						
Mean frequency	Intercept	1	24522.546	0.0000	26995.387	0.0000	14025.858	0.0000
	Genotype	1	0.002	0.9680	4.664	0.0537	0.385	0.5474
	Error	11						
	Treatment	1	0.399	0.5405	2.909	0.1161	0.987	0.3419
	Treatment*Genotype	1	0.000	0.9957	0.444	0.5191	0.101	0.7569
	Error	11						
Power mean frequency	Intercept	1	124.946	0.0000	155.783	0.0000	119.589	0.0000
	Genotype	1	0.036	0.8535	2.579	0.1366	0.184	0.6761
	Error	11						
	Treatment	1	0.250	0.6271	0.092	0.7673	1.428	0.2573
	Treatment*Genotype	1	0.311	0.5885	2.129	0.1725	0.196	0.6664
	Error	11						
Skewness	Intercept	1	11.192	0.0065	16.590	0.0018	31.710	0.0002
	Genotype	1	4.672	0.0536	0.647	0.4381	1.803	0.2064
	Error	11						
	Treatment	1	1.033	0.3313	4.636	0.0543	0.319	0.5836
	Treatment*Genotype	1	0.209	0.6567	0.106	0.7514	0.093	0.7663
	Error	11						
Kurtosis	Intercept	1	43.631	0.0000	139.219	0.0000	136.312	0.0000
	Genotype	1	2.168	0.1690	0.932	0.3550	0.421	0.5299
	Error	11						
	Treatment	1	0.801	0.3900	0.284	0.6045	0.290	0.6011
	Treatment*Genotype	1	0.141	0.7148	0.000	0.9886	0.037	0.8500
	Error	11						

Table S4. Analysis of spectral parameters - hippocampal derivation

Spectral parameters - hippocampal derivation									
Repeated Measures Analysis of Variance									
Waveband	Spectral parameter	Effect	Degr. of (Freedom)	Dose (mg/kg)					
				1		3		10	
				F	P value	F	P value	F	P value
Delta	Band power	Intercept	1	63.208	0.0000	59.577	0.0000	31.694	0.0002
		Genotype	1	2.525	0.1404	1.032	0.3315	0.013	0.9126
		Error	11						
		Treatment	1	0.000	0.9886	0.412	0.5342	0.485	0.5006
		Treatment*Genotype	1	0.498	0.4953	0.013	0.9115	7.556	0.0189
		Error	11						
	Mean frequency	Intercept	1	6044.409	0.0000	6836.831	0.0000	11065.698	0.0000
		Genotype	1	0.306	0.5912	0.140	0.7152	2.162	0.1695
		Error	11						
		Treatment	1	11.199	0.0065	29.988	0.0002	45.008	0.0000
		Treatment*Genotype	1	0.065	0.8032	2.688	0.1294	0.089	0.7705
		Error	11						
	Power mean frequency	Intercept	1	26.947	0.0003	33.266	0.0001	13.286	0.0039
		Genotype	1	3.422	0.0914	0.877	0.3690	0.427	0.5268
		Error	11						
		Treatment	1	1.574	0.2356	3.024	0.1099	3.570	0.0855
		Treatment*Genotype	1	0.049	0.8294	0.042	0.8410	3.551	0.0862
		Error	11						
	Skewness	Intercept	1	98.827	0.0000	97.134	0.0000	202.872	0.0000
		Genotype	1	0.491	0.4981	0.042	0.8411	2.238	0.1628
		Error	11						
		Treatment	1	7.247	0.0210	8.568	0.0138	14.415	0.0030
		Treatment*Genotype	1	3.994	0.0710	11.548	0.0059	2.012	0.1838
		Error	11						

	Kurtosis	Intercept	1	84.540	0.0000	74.242	0.0000	112.861	0.0000
		Genotype	1	0.756	0.4030	0.127	0.7279	0.432	0.5246
		Error	11						
		Treatment	1	5.345	0.0412	4.088	0.0682	9.048	0.0119
		Treatment*Genotype	1	5.345	0.0412	8.270	0.0151	1.606	0.2312
		Error	11						
Theta	Band power	Intercept	1	87.442	0.0000	49.043	0.0000	63.752	0.0000
		Genotype	1	0.297	0.5966	0.619	0.4481	0.496	0.4959
		Error	11						
		Treatment	1	0.403	0.5387	0.003	0.9595	0.118	0.7382
		Treatment*Genotype	1	0.169	0.6885	0.210	0.6554	0.193	0.6687
		Error	11						
	Mean frequency	Intercept	1	2604.848	0.0000	3092.185	0.0000	7848.225	0.0000
		Genotype	1	0.298	0.5961	0.052	0.8241	4.666	0.0537
		Error	11						
		Treatment	1	0.616	0.4493	0.140	0.7154	3.221	0.1002
		Treatment*Genotype	1	0.670	0.4304	0.023	0.8825	6.078	0.0314
		Error	11						
	Power mean frequency	Intercept	1	50.380	0.0000	23.011	0.0006	21.657	0.0007
		Genotype	1	0.343	0.5700	0.154	0.7021	1.383	0.2644
		Error	11						
		Treatment	1	1.603	0.2317	0.069	0.7983	0.073	0.7926
		Treatment*Genotype	1	0.174	0.6849	0.080	0.7822	2.363	0.1525
		Error	11						
	Skewness	Intercept	1	96.296	0.0000	77.922	0.0000	45.053	0.0000
		Genotype	1	1.948	0.1903	0.045	0.8358	0.963	0.3475
		Error	11						
		Treatment	1	1.629	0.2282	1.856	0.2003	8.324	0.0148
		Treatment*Genotype	1	0.486	0.5001	0.662	0.4331	0.845	0.3778
		Error	11						
	Kurtosis	Intercept	1	82.335	0.0000	60.360	0.0000	44.714	0.0000

		Genotype	1	1.170	0.3026	0.023	0.8827	0.172	0.6867
		Error	11						
		Treatment	1	1.762	0.2113	0.867	0.3718	7.960	0.0166
		Treatment*Genotype	1	0.488	0.4992	0.225	0.6444	0.011	0.9190
		Error	11						
Alfa	Band power	Intercept	1	56.765	0.0000	50.126	0.0000	52.908	0.0000
		Genotype	1	2.421	0.1480	0.196	0.6663	0.491	0.4979
		Error	11						
		Treatment	1	1.822	0.2042	0.228	0.6424	0.137	0.7186
		Treatment*Genotype	1	0.097	0.7615	0.020	0.8892	0.464	0.5096
		Error	11						
	Mean frequency	Intercept	1	25476.718	0.0000	11721.016	0.0000	18094.382	0.0000
		Genotype	1	1.494	0.2471	0.124	0.7316	0.103	0.7538
		Error	11						
		Treatment	1	0.025	0.8768	1.211	0.2946	4.140	0.0667
		Treatment*Genotype	1	0.173	0.6852	2.691	0.1292	0.161	0.6961
		Error	11						
	Power mean frequency	Intercept	1	41.518	0.0000	21.052	0.0008	23.277	0.0005
		Genotype	1	0.676	0.4283	0.076	0.7885	1.560	0.2376
		Error	11						
		Treatment	1	1.755	0.2121	0.197	0.6659	0.255	0.6236
		Treatment*Genotype	1	0.166	0.6917	0.020	0.8908	0.793	0.3923
		Error	11						
	Skewness	Intercept	1	21.745	0.0007	73.475	0.0000	38.132	0.0001
		Genotype	1	0.335	0.5743	5.520	0.0385	0.000	0.9982
		Error	11						
		Treatment	1	3.316	0.0959	3.485	0.0888	3.144	0.1039
		Treatment*Genotype	1	0.001	0.9823	2.413	0.1486	0.331	0.5765
		Error	11						
	Kurtosis	Intercept	1	50.433	0.0000	133.952	0.0000	146.134	0.0000
		Genotype	1	0.181	0.6783	4.649	0.0541	1.679	0.2215

		Error	11						
		Treatment	1	3.709	0.0804	1.570	0.2362	0.996	0.3397
		Treatment*Genotype	1	0.120	0.7355	5.421	0.0400	0.864	0.3726
		Error	11						
	Band power	Intercept	1	76.781	0.0000	25.973	0.0003	30.875	0.0002
		Genotype	1	3.909	0.0736	1.180	0.3006	0.076	0.7883
		Error	11						
		Treatment	1	1.509	0.2449	0.074	0.7908	2.398	0.1497
		Treatment*Genotype	1	5.242	0.0428	2.094	0.1758	1.297	0.2790
		Error	11						
	Mean frequency	Intercept	1	125346.473	0.0000	126196.568	0.0000	274331.544	0.0000
		Genotype	1	0.036	0.8531	0.040	0.8450	0.159	0.6981
		Error	11						
		Treatment	1	4.227	0.0643	9.773	0.0096	1.223	0.2924
		Treatment*Genotype	1	0.087	0.7735	0.599	0.4553	0.635	0.4424
		Error	11						
Beta 1	Power mean frequency	Intercept	1	57.956	0.0000	26.704	0.0003	23.023	0.0006
		Genotype	1	3.351	0.0944	1.475	0.2500	0.041	0.8434
		Error	11						
		Treatment	1	0.355	0.5633	0.547	0.4749	1.180	0.3005
		Treatment*Genotype	1	3.394	0.0925	2.246	0.1621	1.725	0.2158
		Error	11						
	Skewness	Intercept	1	5.607	0.0373	0.431	0.5249	4.022	0.0701
		Genotype	1	0.033	0.8597	0.020	0.8909	1.399	0.2619
		Error	11						
		Treatment	1	2.260	0.1609	0.279	0.6080	0.036	0.8535
		Treatment*Genotype	1	0.185	0.6750	0.210	0.6555	2.216	0.1647
		Error	11						
	Kurtosis	Intercept	1	119.841	0.0000	148.961	0.0000	52.540	0.0000
		Genotype	1	0.000	0.9994	0.934	0.3546	2.169	0.1688
		Error	11						

		Treatment	1	0.212	0.6541	0.939	0.3533	0.185	0.6754
		Treatment*Genotype	1	0.184	0.6760	2.128	0.1726	0.643	0.4398
		Error	11						
Beta 2	Band power	Intercept	1	171.091	0.0000	149.793	0.0000	111.484	0.0000
		Genotype	1	0.081	0.7813	1.715	0.2170	1.910	0.1943
		Error	11						
		Treatment	1	2.524	0.1404	0.126	0.7297	0.782	0.3954
		Treatment*Genotype	1	2.442	0.1464	0.878	0.3689	0.782	0.3954
		Error	11						
	Mean frequency	Intercept	1	12899.471	0.0000	3779.477	0.0000	6374.073	0.0000
		Genotype	1	2.963	0.1132	0.008	0.9287	0.355	0.5632
		Error	11						
		Treatment	1	0.763	0.4010	4.720	0.0525	0.174	0.6850
		Treatment*Genotype	1	0.141	0.7145	0.451	0.5156	0.050	0.8264
		Error	11						
	Power mean frequency	Intercept	1	40.109	0.0001	57.017	0.0000	48.520	0.0000
		Genotype	1	0.917	0.3588	4.454	0.0585	0.581	0.4621
		Error	11						
		Treatment	1	5.652	0.0367	3.051	0.1085	2.124	0.1729
		Treatment*Genotype	1	5.644	0.0368	2.113	0.1740	1.151	0.3063
		Error	11						
	Skewness	Intercept	1	27.199	0.0003	39.817	0.0001	39.275	0.0001
		Genotype	1	0.401	0.5393	0.453	0.5150	1.185	0.2996
		Error	11						
		Treatment	1	0.002	0.9619	2.809	0.1219	0.350	0.5663
		Treatment*Genotype	1	1.834	0.2028	0.018	0.8971	0.546	0.4753
		Error	11						
	Kurtosis	Intercept	1	34.189	0.0001	83.220	0.0000	61.742	0.0000
		Genotype	1	0.001	0.9785	1.037	0.3304	0.386	0.5469
		Error	11						
		Treatment	1	1.530	0.2418	1.313	0.2761	3.851	0.0755

		Treatment*Genotype	1	2.450	0.1458	0.010	0.9240	0.014	0.9068
		Error	11						
Gamma	Band power	Intercept	1	271.294	0.0000	79.755	0.0000	127.280	0.0000
		Genotype	1	0.475	0.5050	0.002	0.9622	0.001	0.9753
		Error	11						
		Treatment	1	0.033	0.8600	1.495	0.2470	0.012	0.9135
		Treatment*Genotype	1	0.026	0.8746	0.491	0.4980	0.066	0.8019
		Error	11						
	Mean frequency	Intercept	1	43431.365	0.0000	36429.642	0.0000	20392.661	0.0000
		Genotype	1	0.306	0.5909	0.147	0.7083	1.243	0.2887
		Error	11						
		Treatment	1	0.641	0.4402	0.226	0.6439	2.457	0.1453
		Treatment*Genotype	1	0.003	0.9541	2.685	0.1296	2.995	0.1114
		Error	11						
	Power mean frequency	Intercept	1	71.156	0.0000	44.362	0.0000	67.045	0.0000
		Genotype	1	0.008	0.9300	0.103	0.7540	1.076	0.3218
		Error	11						
		Treatment	1	0.038	0.8493	0.276	0.6100	0.050	0.8267
		Treatment*Genotype	1	0.090	0.7695	0.117	0.7383	0.000	0.9895
		Error	11						
	Skewness	Intercept	1	32.456	0.0001	41.686	0.0000	123.532	0.0000
		Genotype	1	0.964	0.3472	0.394	0.5432	1.161	0.3044
		Error	11						
		Treatment	1	0.621	0.4475	0.001	0.9778	5.063	0.0459
		Treatment*Genotype	1	2.811	0.1218	0.079	0.7845	0.739	0.4085
		Error	11						
	Kurtosis	Intercept	1	24.343	0.0004	124.565	0.0000	92.622	0.0000
		Genotype	1	0.045	0.8363	0.378	0.5512	0.300	0.5947
		Error	11						
		Treatment	1	0.456	0.5136	0.511	0.4895	2.913	0.1159
		Treatment*Genotype	1	2.579	0.1366	0.000	0.9884	0.461	0.5113

		Error	11						
High gamma	Band power	Intercept	1	167.351	0.0000	70.837	0.0000	98.756	0.0000
		Genotype	1	0.000	0.9971	0.044	0.8382	0.677	0.4280
		Error	11						
		Treatment	1	1.710	0.2177	0.020	0.8906	0.050	0.8263
		Treatment*Genotype	1	0.131	0.7242	0.076	0.7873	0.043	0.8390
		Error	11						
	Mean frequency	Intercept	1	15217.484	0.0000	12876.227	0.0000	10474.964	0.0000
		Genotype	1	0.000	0.9845	0.082	0.7801	0.172	0.6863
		Error	11						
		Treatment	1	1.335	0.2724	3.474	0.0892	3.184	0.1019
		Treatment*Genotype	1	0.366	0.5572	4.429	0.0591	1.612	0.2305
		Error	11						
	Power mean frequency	Intercept	1	131.195	0.0000	47.742	0.0000	63.761	0.0000
		Genotype	1	0.155	0.7012	0.253	0.6249	0.285	0.6041
		Error	11						
		Treatment	1	0.750	0.4051	0.001	0.9714	1.214	0.2940
		Treatment*Genotype	1	1.249	0.2876	0.172	0.6860	0.794	0.3919
		Error	11						
	Skewness	Intercept	1	4.485	0.0578	26.929	0.0003	33.830	0.0001
		Genotype	1	0.009	0.9258	1.188	0.2991	2.526	0.1403
		Error	11						
		Treatment	1	1.437	0.2559	4.209	0.0648	2.540	0.1393
		Treatment*Genotype	1	0.012	0.9134	0.807	0.3881	0.601	0.4545
		Error	11						
	Kurtosis	Intercept	1	10.976	0.0069	103.454	0.0000	83.677	0.0000
		Genotype	1	0.039	0.8472	1.306	0.2774	2.114	0.1739
		Error	11						
		Treatment	1	0.857	0.3745	2.976	0.1125	1.869	0.1989
		Treatment*Genotype	1	0.355	0.5635	1.825	0.2038	2.824	0.1210
		Error	11						

Table S5. Fisher's least significant difference (LSD) post hoc analysis - Spectral parameters (cortical and hippocampal derivations)

Spectral parameters								
Fisher's LSD post hoc analysis								
Group/Treatment	Waveband	Spectral parameter	Cortical derivation Dose (mg/kg) (P value)			Hippocampal derivation Dose (mg/kg) (P value)		
			1	3	10	1	3	10
Control (baseline) *vs* Control (treated)	Delta	Band power	np	np	np	np	np	0.0386
		Mean frequency	0.0482	0.0069	0.0150	0.0320	0.0005	0.0011
		Power mean frequency	np	np	np	np	np	np
		Skewness	0.0026	0.0080	0.0010	0.0085	0.0012	0.0045
		Kurtosis	0.0069	0.0177	0.0068	0.0092	0.0066	0.0141
	Theta	Band power	np	np	np	np	np	np
		Mean frequency	0.3159	np	np	np	np	0.0144
		Power mean frequency	0.0230	np	np	np	np	np
		Skewness	0.0006	np	0.1631	np	np	0.0250
		Kurtosis	0.0051	0.1924	np	np	np	0.0716
	Alfa	Band power	np	np	np	np	np	np
		Mean frequency	np	np	np	np	np	np
		Power mean frequency	np	np	np	np	np	np
		Skewness	np	np	np	np	0.0398	np
		Kurtosis	np	np	0.0420	np	0.0328	np
	Beta 1	Band power	0.0456	np	np	0.0354	np	np
		Mean frequency	np	0.1324	np	np	0.1373	np
		Power mean frequency	np	np	np	np	np	np
		Skewness	np	np	0.0670	np	np	np
		Kurtosis	np	np	np	np	np	np

	Beta 2	Band power	0.0037	np	np	np	np	np
		Mean frequency	np	np	np	np	np	np
		Power mean frequency	0.0017	0.0114	np	0.0079	np	np
		Skewness	0.9639	0.0739	np	np	np	np
		Kurtosis	0.6792	np	np	np	np	np
	Gamma	Band power	np	0.1458	0.0459	np	np	np
		Mean frequency	np	np	np	np	np	np
		Power mean frequency	np	np	0.0388	np	np	np
		Skewness	np	np	0.0214	np	np	0.0577
		Kurtosis	np	np	0.0856	np	np	np
Ts65Dn (baseline) *vs* Ts65Dn (treated)	Delta	Band power	np	np	np	np	np	0.1591
		Mean frequency	0.0211	0.0130	0.0017	0.0439	0.0166	0.0003
		Power mean frequency	np	np	np	np	np	np
		Skewness	0.0288	0.1736	0.0012	0.6199	0.7353	0.1078
		Kurtosis	0.2298	0.4985	0.0126	0.9999	0.5425	0.2265
	Theta	Band power	np	np	np	np	np	np
		Mean frequency	0.0089	np	np	np	np	0.6313
		Power mean frequency	0.4766	np	np	np	np	np
		Skewness	0.9066	np	0.1264	np	np	0.1758
		Kurtosis	0.7661	0.0748	np	np	np	0.0708
	Alfa	Band power	np	np	np	np	np	np
		Mean frequency	np	np	np	np	np	np
		Power mean frequency	np	np	np	np	np	np
		Skewness	np	np	np	np	0.8218	np
		Kurtosis	np	np	0.3389	np	0.4454	np
	Beta 1	Band power	0.3311	np	np	0.4514	np	np
		Mean frequency	np	0.0878	np	np	0.0152	np
		Power mean frequency	np	np	np	np	np	np

		Skewness	np	np	0.2004	np	np	np
		Kurtosis	np	np	np	np	np	np
	Beta 2	Band power	0.5804	np	np	np	np	np
		Mean frequency	np	np	np	np	np	np
		Power mean frequency	0.5008	0.9917	np	0.9989	np	np
		Skewness	0.9264	0.0602	np	np	np	np
		Kurtosis	0.5475	np	np	np	np	np
	Gamma	Band power	np	0.1117	0.1839	np	np	np
		Mean frequency	np	np	np	np	np	np
		Power mean frequency	np	np	0.3047	np	np	np
		Skewness	np	np	0.9141	np	np	0.3280
		Kurtosis	np	np	0.8381	np	np	np
Control (baseline) *vs* Ts65Dn (baseline)	Delta	Band power	np	np	np	np	np	0.1712
		Mean frequency	0.5338	0.6402	0.9986	0.7712	0.6359	0.3799
		Power mean frequency	np	np	np	np	np	np
		Skewness	0.6727	0.8400	0.7360	0.5024	0.1036	0.0521
		Kurtosis	0.6041	0.8894	0.8366	0.4493	0.1251	0.1817
	Theta	Band power	np	np	np	np	np	np
		Mean frequency	0.2734	np	np	np	np	0.6863
		Power mean frequency	0.4173	np	np	np	np	np
		Skewness	0.0915	np	0.5886	np	np	0.9283
		Kurtosis	0.3975	0.4712	np	np	np	0.7988
	Alfa	Band power	np	np	np	np	np	np
		Mean frequency	np	np	np	np	np	np
		Power mean frequency	np	np	np	np	np	np
		Skewness	np	np	np	np	0.8888	np
		Kurtosis	np	np	0.1750	np	0.7095	np
	Beta 1	Band power	0.1145	np	np	0.6077	np	np
		Mean frequency	np	0.5127	np	np	0.8065	np

		Power mean frequency	np	np	np	np	np	np
		Skewness	np	np	0.2484	np	np	np
		Kurtosis	np	np	np	np	np	np
	Beta 2	Band power	0.7948	np	np	np	np	np
		Mean frequency	np	np	np	np	np	np
		Power mean frequency	0.8043	0.6343	np	0.6046	np	np
		Skewness	0.0260	0.5833	np	np	np	np
		Kurtosis	0.0508	np	np	np	np	np
	Gamma	Band power	np	0.2171	0.9693	np	np	np
		Mean frequency	np	np	np	np	np	np
		Power mean frequency	np	np	0.9162	np	np	np
		Skewness	np	np	0.4357	np	np	0.8351
		Kurtosis	np	np	0.4761	np	np	np
Control (treated) *vs* Ts65Dn (treated)	Delta	Band power	np	np	np	np	np	0.2348
		Mean frequency	0.7019	0.2987	0.3032	0.5572	0.2724	0.2089
		Power mean frequency	np	np	np	np	np	np
		Skewness	0.3806	0.0480	0.8688	0.0888	0.0542	0.9572
		Kurtosis	0.1815	0.0395	0.6939	0.0455	0.0441	0.6262
	Theta	Band power	np	np	np	np	np	np
		Mean frequency	0.0610	np	np	np	np	0.0035
		Power mean frequency	0.7533	np	np	np	np	np
		Skewness	0.0056	np	0.5588	np	np	0.1925
		Kurtosis	0.0067	0.7845	np	np	np	0.7017
	Alfa	Band power	np	np	np	np	np	np
		Mean frequency	np	np	np	np	np	np
		Power mean frequency	np	np	np	np	np	np
		Skewness	np	np	np	np	0.0155	np

	Kurtosis	np	np	0.1398	np	0.0045	np
	Band power	0.0237	np	np	0.0102	np	np
	Mean frequency	np	0.5833	np	np	0.5651	np
Beta 1	Power mean frequency	np	np	np	np	np	np
	Skewness	np	np	0.0788	np	np	np
	Kurtosis	np	np	np	np	np	np
	Band power	0.0864	np	np	np	np	np
	Mean frequency	np	np	np	np	np	np
Beta 2	Power mean frequency	0.0017	0.0739	np	0.0474	np	np
	Skewness	0.0276	0.5640	np	np	np	np
	Kurtosis	0.0428	np	np	np	np	np
	Band power	np	0.2339	0.4267	np	np	np
	Mean frequency	np	np	np	np	np	np
Gamma	Power mean frequency	np	np	0.3500	np	np	np
	Skewness	np	np	0.0007	np	np	0.2170
	Kurtosis	np	np	0.0053	np	np	np

Table S6. Analysis of theta-burst stimulation (TBS)-induced long-term potentiation (LTP) levels

TBS-induced LTP levels				
One-way Analysis of Variance				
Group	Effect	Degr. of (Freedom)	F	P value
Control	Intercept	1	1254.084	0.0000
	Treatment	3	4.103	0.0119
	Error	44		
Ts65Dn	Intercept	1	732.178	0.0000
	Treatment	3	0.857	0.8570
	Error	44		

Table S7. Fisher's least significant difference (LSD) post hoc analysis - TBS-induced LTP levels

TBS-induced LTP levels	
Fisher's LSD post hoc analysis	
Treament	P value
GABA 200 µM	0.0489
Diazepam 1 µM	0.0458
Diazepam 10 µM	0.0011

Table S8. Analysis of normalized theta-burst stimulation (TBS)-induced long-term potentiation (LTP) levels

Normalized TBS-induced LTP levels				
Two-way Analysis of Variance				
Parameter	Effect	Degr. of (Freedom)	F	P value
TBS-induced LTP levels	Intercept	1	1764.961	0.0000
	Genotype	1	10.987	0.0013
	Treatment	3	1.494	0.2217
	Genotype*Treatment	3	1.314	0.2750
	Error	88		

Table S9. Fisher's least significant difference (LSD) post hoc analysis - Normalized TBS-induced LTP levels

Normalized TBS-induced LTP levels	
Fisher's LSD post hoc analysis	
Group/Treatment	P value
Control (aCSF) *vs* Ts65Dn (aCSF)	0.9999
Control (GABA 200 µM) *vs* Ts65Dn (GABA 200 µM)	0.0211
Control (Diazepam 1 µM) *vs* Ts65Dn (Diazepam 1 µM)	0.0775
Control (Diazepam 10 µM) *vs* Ts65Dn (Diazepam 10 µM)	0.0145

Table S10. Analysis of Input-Output (I/O) and Paired-pulse facilitation (PPF)

I/O and PPF				
Repeated Measures Analysis of Variance				
Group/Parameter	Effect	Degr. of (Freedom)	F	P value
Control (I/O Curve)	Intercept	1	156.688	0.0000
	Treatment	3	0.113	0.9523
	Error	44		
	Stimulus	9	147.788	0.0000
	Stimulus*Treatment	27	0.238	0.9999
	Error	396		
Ts65Dn (I/O Curve)	Intercept	1	282.667	0.0000
	Treatment	3	0.416	0.7425
	Error	44		
	Stimulus	9	228.287	0.0000
	Stimulus*Treatment	27	0. 258	0.9999
	Error	396		
Control (PPF)	Intercept	1	5377.232	0.0000
	Treatment	3	0.838	0.4802
	Error	44		
	Stimulus	7	365.632	0.0000
	Stimulus*Treatment	21	1.265	0.1967
	Error	308		
Ts65Dn (PPF)	Intercept	1	4962.442	0.0000
	Treatment	3	0.36	0.7824
	Error	44		
	Stimulus	7	336.41	0.0000
	Stimulus*Treatment	21	0.95	0.5261
	Error	308		

Table S11. Analysis of susceptibility to picrotoxin-induced seizures

Susceptibility to picrotoxin-induced seizures					
Two-way Analysis of Variance					
Treatment	Seizure parameter	Effect	Degr. of (Freedom)	F	P value
2 mg/kg PTX *vs* 4 mg/kg PTX	Seizure severity	Intercept	1	432.704	0.0000
		Genotype	1	0.295	0.5892
		Treatment	1	30.002	0.0000
		Genotype*Treatment	1	0.933	0.3389
		Error	50		
	Latency	Intercept	1	298.764	0.0000
		Genotype	1	1.247	0.2713
		Treatment	1	16.275	0.0003
		Genotype*Treatment	1	0.035	0.8537
		Error	37		
3 mg/kg DZP pretreatment *vs* no pretreatment	Seizure severity	Intercept	1	105.356	0.0000
		Genotype	1	0.393	0.5333
		Treatment	1	16.668	0.0002
		Genotype*Treatment	1	0.617	0.4356
		Error	52		

Table S12. Fisher's least significant difference (LSD) post hoc analysis – Seizure parameters

Susceptibility to picrotoxin-induced seizures		
Fisher's LSD post hoc analysis		
Group/Treatment	Seizure parameter	P value
Control (2 mg/kg PTX) *vs* Control (4 mg/kg PTX)	Seizure severity	0.0035
	Latency	0.0089
Ts65Dn (2 mg/kg PTX) *vs* Ts65Dn (4 mg/kg PTX)	Seizure severity	0.0000
	Latency	0.0056
Control (2 mg/kg PTX) *vs* Ts65Dn (2 mg/kg PTX)	Seizure severity	0.2710
	Latency	0.5548
Control (4 mg/kg PTX) *vs* Ts65Dn (4 mg/kg PTX)	Seizure severity	0.7752
	Latency	0.3037
3 mg/kg DZP pretreated control *vs* non-pretreated control	Seizure severity	0.0016
3 mg/kg DZP pretreated Ts65Dn *vs* non-pretreated Ts65Dn	Seizure severity	0.0189
Non-pretreated control *vs* non-pretreated Ts65Dn	Seizure severity	0.3123
3 mg/kg DZP pretreated control *vs*3 mg/kg DZP pretreated control	Seizure severity	0.9130

Table S13. Analysis of spatiotemporal and ethological parameters - Elevated plus-maze behavioral test

Analysis of spatiotemporal and ethological parameters - Elevated plus-maze behavioral test				
Two-way Analysis of Variance				
Parameter	Effect	Degr. of (Freedom)	F	P value
Open arm entries	Intercept	1	71.156	0.0000
	Genotype	1	3.935	0.0522
	Treatment	1	6.123	0.0164
	Genotype*Treatment	1	1.065	0.3066
	Error	56		
Time spent in open arms	Intercept	1	60.136	0.0000
	Genotype	1	2.337	0.1319
	Treatment	1	10.124	0.0024
	Genotype*Treatment	1	0.290	0.5921
	Error	56		
Closed arm entries	Intercept	1	131.858	0.0000
	Genotype	1	0.077	0.7818
	Treatment	1	3.831	0.0553
	Genotype*Treatment	1	0.127	0.7227
	Error	56		
Time spent in closed arms	Intercept	1	304.666	0.0000
	Genotype	1	0.980	0.3264
	Treatment	1	1.041	0.3119
	Genotype*Treatment	1	0.004	0.9525
	Error	56		
Total arms entries	Intercept	1	124.768	0.0000
	Genotype	1	1.412	0.2397
	Treatment	1	5.947	0.0179
	Genotype*Treatment	1	0.553	0.4600
	Error	56		

Time spent in the center	Intercept	1	173.652	0.0000
	Genotype	1	0.111	0.7404
	Treatment	1	3.920	0.0526
	Genotype*Treatment	1	0.501	0.4822
	Error	56		
Protected stretched attend posture frequency	Intercept	1	248.198	0.0000
	Genotype	1	0.551	0.4611
	Treatment	1	6.955	0.0108
	Genotype*Treatment	1	5.104	0.0278
	Error	56		
Unprotected stretched attend posture frequency	Intercept	1	35.981	0.0000
	Genotype	1	0.071	0.7903
	Treatment	1	7.253	0.0093
	Genotype*Treatment	1	1.721	0.1949
	Error	56		
Protected head dipping frequency	Intercept	1	185.143	0.0000
	Genotype	1	4.121	0.0471
	Treatment	1	18.087	0.0001
	Genotype*Treatment	1	1.058	0.3082
	Error	56		
Unprotected head dipping frequency	Intercept	1	125.429	0.0000
	Genotype	1	1.214	0.2752
	Treatment	1	17.866	0.0001
	Genotype*Treatment	1	1.045	0.3111
	Error	56		
Closed arm return frequency	Intercept	1	7.421	0.0086
	Genotype	1	5.833	0.0190
	Treatment	1	1.529	0.2214
	Genotype*Treatment	1	0.860	0.3577
	Error	56		
Grooming time	Intercept	1	52.012	0.0000
	Genotype	1	6.359	0.0145

Treatment	1	2.224	0.1415
Genotype*Treatment	1	0.025	0.8748
Error	56		

Table S14. Fisher's least significant difference (LSD) post hoc analysis - Elevated plus-maze behavioral parameters

Analysis of spatiotemporal and ethological parameters - Elevated plus-maze behavioral test		
Fisher's LSD post hoc analysis		
Genotype	Behavioral parameter	P value
Control (saline) *vs* Control (treated)	Open arm entries	0.0130
	Time spent in open arms	0.0086
	Total arms entries	0.0235
	Protected stretched attend posture frequency	0.0007
	Unprotected stretched attend posture frequency	0.0049
	Protected head dipping frequency	0.0003
	Unprotected head dipping frequency	0.0003
	Closed arm return frequency	0.1189
	Grooming time	0.3334
Ts65Dn (saline) *vs* Ts65Dn (treated)	Open arm entries	0.3275
	Time spent in open arms	0.0757
	Total arms entries	0.2509
	Protected stretched attend posture frequency	0.7967
	Unprotected stretched attend posture frequency	0.3484
	Protected head dipping frequency	0.0314
	Unprotected head dipping frequency	0.0324
	Closed arm return frequency	0.8331
	Grooming time	0.2635
Control (saline) *vs* Ts65Dn (saline)	Open arm entries	0.0374
	Time spent in open arms	0.1493
	Total arms entries	0.1773
	Protected stretched attend posture frequency	0.0383
	Unprotected stretched attend posture frequency	0.2690
	Protected head dipping frequency	0.0349

	Unprotected head dipping frequency	0.1387
	Closed arm return frequency	0.0216
	Grooming time	0.1003
Control (treated) *vs* Ts65Dn (treated)	Open arm entries	0.5037
	Time spent in open arms	0.4868
	Total arms entries	0.7546
	Protected stretched attend posture frequency	0.2880
	Unprotected stretched attend posture frequency	0.4632
	Protected head dipping frequency	0.4818
	Unprotected head dipping frequency	0.9552
	Closed arm return frequency	0.2973
	Grooming time	0.0633
